# Microalgae as a New Source of Oxylipins: A Comprehensive LC-MS-Based Analysis Using Conventional and Green Extraction Methods

**DOI:** 10.1021/acs.jafc.4c03264

**Published:** 2024-07-17

**Authors:** Arturo Auñon-Lopez, Jon Alberdi-Cedeño, Marc Pignitter, Natalia Castejón

**Affiliations:** † Institute of Physiological Chemistry, Faculty of Chemistry, University of Vienna, 1090 Vienna, Austria; ‡ Vienna Doctoral School in Chemistry (DoSChem), University of Vienna, Währinger Str. 42, 1090 Vienna, Austria; § Food Technology, Faculty of Pharmacy, Lascaray Research Center, University of the Basque Country (UPV/EHU), Paseo de la Universidad 7, 01006 Vitoria-Gasteiz (Alava), Spain; ∥ Institute of Food Chemistry and Toxicology, Faculty of Chemistry, University of Vienna, Waehringer Str. 38, 1090 Vienna, Austria

**Keywords:** microalgal lipids, polyunsaturated fatty acids, oxylipins, liquid chromatography−mass spectrometry, eco-friendly methods

## Abstract

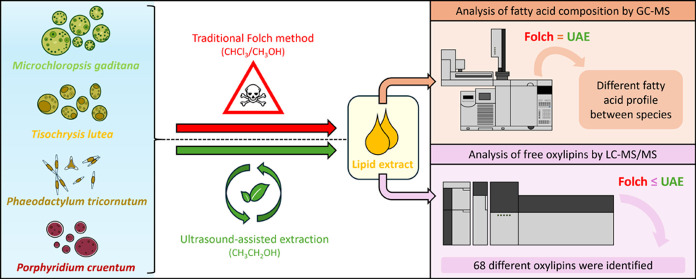

Microalgae are promising sources of essential lipids, including omega-3 and omega-6 polyunsaturated fatty acids (n-3 and n-6 PUFA) and novel lipid metabolites like oxylipins. However, limited data exist on the oxylipin profile, its characterization, and the potential impact of the extraction process on these metabolites in microalgae. Thus, our study aimed to investigate the fatty acid and oxylipin profile of four microalgal species of interest (*Microchloropsis gaditana*, *Tisochrysis lutea*, *Phaeodactylum tricornutum*, and *Porphyridium cruentum*) while also examining the impact of the extraction method, with a focus on developing a greener process using ultrasound-assisted extraction (UAE) and ethanol. The UAE method showed similar oxylipin profiles, generally yielding concentrations comparable to those of the conventional Folch method. In total, 68 oxylipins derived from n-3 and n-6 PUFA were detected, with the highest concentrations of n-3 oxylipins found in *P. tricornutum* and *T. lutea* and of n-6 oxylipins in *P. cruentum*. This study provides the most extensive oxylipin characterization of these microalgae species to date, offering insights into alternative extraction methods and opening new avenues for further investigation of the significance of oxylipins in microalgae.

## 1 Introduction

The demand for sustainable and natural food ingredients, driven by increasing consumer interest, has experienced outstanding growth in recent years. In this sense, microalgae have emerged as a highly suitable option for meeting population needs concerning valuable lipids, including omega-3 and omega-6 polyunsaturated fatty acids (n-3 and n-6 PUFA), proteins, and other minor compounds such as carotenoids and chlorophylls.^1−4^ Regarding lipids, cultured microalgae have emerged as an environmentally friendly and sustainable alternative to fish oil for obtaining n-3 fatty acids in the human diet.^5^ As primary producers of long-chain n-3 PUFA, namely, eicosapentaenoic (EPA) and docosahexaenoic (DHA) acids, microalgae offer several advantages. Microalgae cultivation not only relieves pressure on wild fish stocks but also contributes to environmental sustainability by reducing the ecological impact associated with traditional fish oil production.^6^ Moreover, from a nutritional perspective, microalgae produce a wide range of high-value lipids, including the already mentioned n-3 and n-6 PUFA, neutral lipids, and more complex lipids such as phospholipids and glycolipids,^7^ making them ideal candidates for applications in the food and pharmaceutical industries.

The extraction of lipids from microalgae remains challenging, primarily due to the limited accessibility caused by the complexity of the cell membrane and the rigidity of the cell wall.^8^ Extraction methods traditionally employed for marine lipids include the chloroform- and methanol-based Folch method. However, recent trends emphasize the growing necessity for greener approaches. For instance, ultrasound-assisted extraction (UAE) is an emerging technology that disrupts cells primarily through cavitation and acoustic effects, resulting in an enhanced lipid extraction rate.^9^ Compared to classical methods, UAE requires less energy, is faster, more efficient, and enables the use of environmentally friendly solvents such as ethanol.^10^ UAE has been successfully employed in recent years for extracting microalgal lipids.^11−14^

A group of compounds that could be found in the lipidic fraction of microalgae are oxylipins. Besides mammals, marine algae, including seaweeds and microalgae, are recognized for their ability to biosynthesize these compounds.^15,16^ Oxylipins are molecules derived from either enzymatically or nonenzymatically oxidation of PUFA, which include fatty acids with hydroperoxy-, hydroxy-, oxo- or epoxy-functional groups, among others.^17^ Most of them, especially those derived from n-6 and n-3 PUFA, such as arachidonic acid (ARA), EPA, and DHA, play a crucial role as significant lipid mediators in the human body, regulating processes such as inflammation, pain, blood pressure, or renal function.^18^ Oxylipins have been recognized as biomolecules with promising therapeutic applications due to their anticancer, anti-inflammatory, and antimicrobial properties.^19^ Additionally, other oxylipins, derived mostly from linoleic acyl groups, can play beneficial or deleterious roles in diseases such as cancer, Alzheimer’s, Parkinson’s, acute respiratory distress syndrome (ARDS), circulatory shock, disseminated intravascular coagulation and multiple organ failure, among others.^20−22^ It should be pointed out that the oxylipins circulating in human plasma can result from either endogenous formation of PUFA or the diet.^23,24^

However, the available data on the oxylipin profile, their comprehensive characterization, and the potential impact of the extraction process on these biomolecules, particularly in microalgae, are limited, and the research field is still emerging. For instance, the detection of the EPA-derived metabolite 15(S)-hydroxyeicosapentaenoic acid (15(S)-HEPE) was reported for the first time in the microalga species *Microchloropsis gaditana* (formerly *Nannochloropsis gaditana*), known for its high lipid and EPA content.^25^ In the same study, the authors reported a wide range of oxylipins in the cultures of the freshwater species *Chlamydomonas debaryana*. The same research group described the biological activity of oxylipins derived from these two microalgal species in terms of anti-inflammatory and cytotoxic activity.^25−27^ Only a few additional accounts of oxylipins from microalgae have been reported, including an isoprostanoid profile in some marine species,^28^ the exploration of marine diatoms,^29−31^ and some commercial algae oil supplements.^24,32^ More recently, Linares-Maurizi et al. stated the oxylipin profile of five microalgae species (not specified in their study), revealing a high diversity of these metabolites in marine matrices.^33^ While this limited amount of studies have emphasized the importance of research on microalgal oxylipins, many species remain uncharacterized despite their potential as oxylipin sources.

Therefore, in this study, we investigated the fatty acid profile and oxylipin pattern in four microalgae species rich in n-3 and n-6 PUFA using gas chromatography–mass spectrometry (GC-MS) and liquid chromatography–tandem mass spectrometry (LC-MS/MS), respectively. We hypothesized that due to the high PUFA content, microalgae can serve as a reservoir of new bioactive oxylipins. Microalgae species used herein were selected to cover a wide range of PUFA, with a particular focus on n-3 and n-6 fatty acids. Additionally, we examined the impact of the extraction method on the fatty acid and oxylipin profiles; specifically, we compared the classical Folch method with a greener approach using UAE and ethanol. Our study provides novel insights into the oxylipin pattern in microalgal lipid extracts, which could open new avenues for extensive research in this field, and, for the first time, explores the impact of the extraction method on these specific metabolites.

## 2 Materials and Methods

### 2.1 Materials

The spray-dried microalgal biomass of *M. gaditana* (batch 02092021Ng), *Tisochrysis lutea* (batch 02092021TL), *Phaeodactylum tricornutum* (batch 02092021Pt), and *Porphyridium cruentum* (batch 02092021Pc) was purchased from Cianoalgae SI (Gipuzkoa, Spain).

Oxylipin standards, namely, 15(S)-HEPE-*d*_5_, 17(S)-Resolvin D1-*d*_5_, 13-oxo-octadecadienoic acid-d_3_ (13-oxo-ODE-*d*_3_), 17(S)-hydroperoxydocosahexaenoic acid (17-HpDHA), prostaglandin E_3_ (PGE_3_), leukotriene B_5_ (LTB_5_), and 17,18-epoxyeicosatetraenoic acid (17,18-EpETE), were all purchased from Cayman Chemicals (Ann Arbor, MI). Fatty acid methyl esters standard (Supelco 37 FAME Mix) was from Supelco (Bellefonte, PA). Chloroform, methanol, and ethanol were purchased from Fisher Scientific GmbH (Vienna, Austria). Ethyl acetate, sodium hydrogen carbonate, and potassium hydroxide were acquired from Carl Roth GmbH & Co. (Karlsruhe, Germany). LC-MS grade solvents and additives (water, acetonitrile, methanol, and acetic acid) were purchased from Avantor/VWR International, Inc. (Radnor, PA).

### 2.2 Lipid Extraction of Microalgal Biomass

#### 2.2.1 Folch

The Folch extraction method was done following the original procedure.^34^ A total of 1 g of microalgal biomass was extracted with 20 mL of chloroform:methanol (2:1) vortexing for 2 min. The mixture was centrifuged at 3000 rpm for 10 min, and the organic layer was collected. The extraction process was carried out 3 times on the same biomass. The collected organic layers were purified by washing them with water and centrifuged at 4000 rpm for 10 min. Finally, the chloroform layer contained the extracted lipids. Samples were evaporated in a centrifugal vacuum concentrator (CentriVap Complete Vacuum Concentrator, Labconco). Lipid extracts were stored in dark vessels with an argon atmosphere at 4 °C until their analysis.

#### 2.2.2 Ultrasound-Assisted Extraction

UAE was carried out with an ultrasound bath (Elmasonic P 30H, Elma Schmidbauer GmbH, Singen, Germany) with automatic control of the time and temperature. Extractions were done using an ultrasound frequency of 37 kHz, and ultrasonic power of 100 W. Dried microalgal biomass was dispersed in ethanol at a 1:10 (w/v) ratio and extracted for 30 min at 30 °C. After the treatment, samples were filtrated, evaporated, and treated as previously described for the Folch extraction method.

### 2.3 Fatty Acid Composition by GC-MS

Fatty acid composition of all microalgal extracts was analyzed by GC–MS using an Agilent 7890A connected to an Agilent 5975C Inert XL EI/CI MSD (Palo Alto, CA). Before analysis, fatty acid methyl esters (FAMEs) were freshly prepared by base-catalyzed methanolysis of the glycerides (KOH in methanol). FAMEs were separated by using an HP-5 ms ultrainert column (30 m × 250 μm × 0.25 μm) (Palo Alto, CA). A total of 1 μL of the sample was injected in splitless mode, and the injector temperature was 280 °C. The initial oven temperature was set at 150 °C for 1 min, and the temperature was gradually raised to 220 °C at 3 °C/min with a final increase to 300 °C for 3 min. Helium was used as the carrier gas at a constant column flow rate of 2.52 mL/min. The GC-MS interface temperature was fixed at 280 °C, and the mass analyzer was set in scan mode. The mass range evaluated was 50–600 *m*/*z*, where the MS quad and source temperatures were maintained at 150 and 230 °C, respectively. Fatty acids were identified by comparing their retention times and mass spectrum profiles with known standards (FAME mix supelco) and the NIST mass spectral library (Version 2.2).

### 2.4 Sample Preparation for LC-MS/MS Analysis by Solid Phase Extraction (SPE)

In order to purify the free oxylipins from the microalgae lipid extracts, an SPE was performed based on a previous study,^32^ with some modifications. In short, the SPE cartridge, Strata-X 33 μm Polymeric Reversed Phase, 30 mg/1 mL tube (Phenomenex, Torrance) was washed once with 1 mL of ethyl acetate, twice with 1 mL of methanol, and then conditioned with 2 × 1 mL of buffer (5% methanol in water with 0.1% acetic acid). A total of 30 mg of lipid extract was then diluted in 1 mL of ethyl acetate and 2 μL of each deuterated standard (Section 2.1) were added to a final concentration of 0.2 μg/mL. The sample was then loaded into the cartridge, and the part of the sample running through the column was collected and later unified with the eluted sample at the end. The cartridge was subsequently washed twice with 1 mL of buffer. After drying the cartridge 20 min under vacuum, the free oxylipins were eluted from the cartridge with 1 mL of methanol and 3 × 1 mL of ethyl acetate. The samples were dried under nitrogen and reconstituted with 300 μL of methanol. After filtering them with Rotilabo PVDF 15 mm Syringe Filters 0.2 μm (Carl Roth GmbH & CO., Karlsruhe, Germany), the samples were analyzed by LC-MS/MS.

### 2.5 LC-MS/MS Analysis of Free Oxylipins

To determine the free oxylipins present in the microalgae extracts, samples (10 μL) were injected into an LC-20 system with an LCMS-8040 detector (Shimadzu, Korneuburg, Austria). The free oxylipins were separated by the LC-20 using a C12 column (Synergi 4 μm Max-RP 80 Å, 150 × 2 mm, Phenomenex, Torrance, CA). The mobile phase was water with 0.1% acetic acid (solvent A) and acetonitrile/methanol (80:15) with 0.1% acetic acid (solvent B). The following gradient was applied: 0–1 min 25% B, 1–1.5 min 25 to 30% B, 1.5–10 min 30 to 53% B, 10–19.5 min 53 to 68% B, 19.5–24.5 min 68 to 95% B, 24.5–34.5 min 95% B, 34.5–35 min 95 to 25%B, 35–38.5 min 25% B. The flow rate was 0.3 mL/min, and the oven temperature was 25 °C. MS analysis was performed in an electrospray ionization (ESI) triple quadrupole mass spectrometer in negative mode, with the following ESI ion source settings: nebulizing gas flow 3 L/min (N_2_), drying gas flow 10 L/min (N_2_), desolvation line temperature 150 °C, and heat block temperature 350 °C. MS/MS analysis was performed in multiple reaction monitoring (MRM) mode, with argon as collision-induced dissociation (CID) gas. The list of the different MRM transitions with their corresponding collision energies can be found in Table S1.

Data processing was performed with LabSolutions software version 5.99 SP2 (Shimadzu, Korneuburg, Austria) and Skyline version 22.1 by MacCoss Lab Software.^35^ Oxylipin quantification was carried out using each standard as representative of one oxylipin class: deuterated versions were used as internal standards and, when not available, nondeuterated versions as external standards. The limits of detection (LOD) and quantification (LOQ) were determined by signal-to-noise ratios of 3 and 10, respectively.

### 2.6 Statistical Analysis

All extractions were performed at least in triplicate (*n* ≥ 3). The effect of extraction method on the fatty acid composition was analyzed by using a one-way analysis of variance (ANOVA) followed by a Tukey post hoc test. For the comparison on the oxylipin profile, differences between microalgal species were analyzed by one-way ANOVA followed by a Tukey post hoc test as well, while the effect of the extraction method on the oxylipin content was evaluated by unpaired two-tailed *t* test. Normal distribution and equal variance were assessed by Shapiro–Wilk and Brown–Forsythe tests, respectively. When variances were significantly different, Welch ANOVA followed by Dunnett T3 post hoc test or unpaired *t* test with Welch’s correction were performed. Differences were considered statistically significant at *p* < 0.05, *p* < 0.01, and *p* < 0.001 for comparisons between extraction methods, whereas a significance level of *p* < 0.05 was used for comparisons between microalgae species.

## 3 Results and Discussion

### 3.1 Fatty Acid Profile of Microalgal Lipid Extracts

Table 1 shows the fatty acid composition (as percentages of total fatty acids) of lipid extracts obtained from *M. gaditana*, *T. lutea*, *P. tricornutum*, and *P. cruentum*, comparing the ultrasound approach with the traditional Folch method. In general, the microalgal lipid extracts were characterized by a high percentage of PUFA (36–63%) and a low n-6/n-3 ratio (0.1–1.6), showing the excellent nutritional properties of these particular microalgae species. Among the PUFA, the abundance of n-3 PUFA (31–58%) is noteworthy, especially in *M. gaditana*, *T. lutea*, and *P. tricornutum*, while *P. cruentum* extracts were characterized for a higher n-6 PUFA (31–33%). Regarding the specific fatty acid composition, with a focus on n-3 and n-6 PUFA, the investigated microalgae species exhibited different profiles. For instance, in *M. gaditana* extracts, eicosapentaenoic acid (EPA; 20:5 *all-cis*-5,8,11,14,17) was the main n-3 PUFA, ranging from 41 to 42%. In *T. lutea* extracts, the n-3 PUFA α-linolenic acid (ALA; 18.3 *all-cis*-9,12,15), stearidonic acid (SDA, 18:4 *all-cis*-6,9,12,15), and docosahexaenoic acid (DHA, 22:6 *all-cis*-4,7,10,13,16,19) were identified with a relative abundance range of 11–12, 32–33, and 12–13%, respectively. EPA was also the main n-3 PUFA in *P. tricornutum* extracts (17–18%), with a high percentage of hexadecatrienoic acid (HTA, 16:3 *all-cis*-7,10,13) (13%) and a low content of DHA (0.8–0.9%). For *P. cruentum*, the extracts were characterized by the n-3 EPA (21%) and the n-6 PUFA linoleic acid (LA, 18:2 *all-cis*-9,12) (6%) and arachidonic acid (ARA, 20:6 all-cis-5,8,11,14) (21%). In general, the fatty acid profile of all microalgae extracts aligns with previous findings.^11,36−39^ However, it is worth mentioning that the fatty acid profile of microalgae could vary depending on the growing conditions,^40,41^ which sometimes makes their comparison challenging.

**Table 1:** Fatty Acid Composition of Microalgae Lipid Extracts Determined by GC–MS Using the Traditional Folch Method and Ultrasound-Assisted Extraction (UAE)^a^

	% fatty acids							
	*M. gaditana*		*T. lutea*		*P. tricornutum*		*P. cruentum*	
fatty acid	Folch	UAE	Folch	UAE	Folch	UAE	Folch	UAE
14:0	4.2 ± < 0.1^b^	4.4 ± 0.1^a^	17.6 ± 0.6^b^	18.7 ± 0.4^a^	8.0 ± 0.2^a^	7.8 ± 0.3^a^	n.d.	n.d.
15:0	n.d.	n.d.	0.8 ± 0.1^a^	0.9 ± 0.1^a^	n.d.	n.d.	n.d.	n.d.
16:0	20.7 ± 0.1^a^	19.5 ± 0.5^b^	11.6 ± 0.4^b^	13.9 ± 1.1^a^	24.9 ± 0.5^b^	25.8 ± 0.2^a^	44.3 ± 0.5^b^	45.7 ± 0.4^a^
16:1n-7	21.6 ± 0.2^a^	22.0 ± 0.5^a^	7.2 ± 0.1^a^	6.9 ± 0.1^b^	25.7 ± 0.2^a^	25.7 ± 0.5^a^	n.d.	n.d.
16:3n-3	n.d.	n.d.	n.d.	n.d.	12.7 ± 0.8^a^	13.4 ± 0.3^a^	n.d.	n.d.
16:4	n.d.	n.d.	n.d.	n.d.	1.5 ± 0.1^b^	2.1 ± 0.1^a^	n.d.	n.d.
18:0	n.d.	n.d.	n.d.	n.d.	n.d.	n.d.	0.5 ± < 0.1^a^	0.4 ± < 0.1^a^
18:1n-7	0.6 ± < 0.1^a^	0.7 ± < 0.1^a^	n.d.	n.d.	1.1 ± < 0.1^b^	1.4 ± < 0.1^a^	0.8 ± 0.2^a^	0.9 ± < 0.1^a^
18:1n-9	3.9 ± 0.2^a^	3.9 ± 0.1^a^	n.d.	n.d.	3.4 ± 0.1^a^	3.1 ± 1.0^b^	0.8 ± 0.2^a^	1.0 ± 0.1^a^
18:2n-6	3.6 ± 0.2^a^	3.7 ± 0.1^a^	3.6 ± 0.1^a^	3.5 ± 0.1^a^	2.3 ± < 0.1^a^	2.2 ± < 0.1^a^	6.4 ± 0.2^a^	6.4 ± 0.1^a^
18:3n-3	n.d.	n.d.	12.1 ± 0.5^a^	10.8 ± 0.3^b^	n.d.	n.d.	n.d.	n.d.
18:4n-3	n.d.	n.d.	32.7 ± 1.3^a^	31.8 ± 1.4^a^	n.d.	n.d.	n.d.	n.d.
20:2n-6	n.d.	n.d.	n.d.	n.d.	n.d.	n.d.	1.9 ± 0.1^a^	1.6 ± 0.1^b^
20:3n-6	n.d.	n.d.	n.d.	n.d.	n.d.	n.d.	0.9 ± 0.1^a^	0.7 ± < 0.1^b^
20:4n-6	4.0 ± 0.1^a^	3.2 ± 0.1^b^	n.d.	n.d.	0.6 ± < 0.1^a^	0.6 ± < 0.1^a^	23.7 ± 0.3^a^	22.7 ± < 0.1^a^
20:5n-3	41.5 ± 0.4^a^	42.2 ± 0.7^a^	n.d.	n.d.	18.5 ± 0.8^a^	16.9 ± 0.4^b^	20.8 ± 0.3^a^	20.8 ± 0.4^a^
22:0	n.d.	n.d.	n.d.	n.d.	0.2 ± < 0.1^a^	0.2 ± 0.2^a^	n.d.	n.d.
22:5n-6	n.d.	n.d.	1.4 ± 0.2^a^	1.4 ± 0.3^a^	n.d.	n.d.	n.d.	n.d.
22:6n-3	n.d.	n.d.	13.0 ± 1.1^a^	12.1 ± 0.5^a^	0.9 ± 0.1^a^	0.8 ± < 0.1^b^	n.d.	n.d.
SFA	24.9 ± 0.1^a^	24.2 ± 0.4^b^	30.0 ± 0.1^b^	33.4 ± 1.4^a^	33.2 ± 0.6^a^	33.8 ± 0.2^a^	44.8 ± 0.4^a^	46.0 ± 0.4^a^
MUFA	26.1 ± 0.1^a^	26.7 ± 0.4^a^	7.2 ± 0.1^a^	6.9 ± 0.1^b^	30.2 ± 0.2^a^	30.2 ± 0.6^a^	1.2 ± < 0.1^a^	1.5 ± 0.4^a^
PUFA	49.0 ± 0.1^a^	49.1 ± 0.7^a^	62.8 ± 0.2^a^	59.7 ± 1.4^b^	36.6 ± 0.8^a^	36.1 ± 0.5^a^	53.6 ± 0.4^a^	52.2 ± 0.5^b^
n-3	41.5	42.2	57.9	54.8	32.1	31.1	20.8	20.8
n-6	10.6	10.7	4.9	4.9	2.9	2.8	32.8	31.3
n-6/n-3 ratio	0.3	0.3	0.1	0.1	0.1	0.1	1.6	1.5

a Results are expressed as a percentage of the total content (relative content). Data is shown as mean ± SD (*n* = 3). Different letters indicate statistically significant differences between extraction methods for each species at *p* < 0.05 (one-way ANOVA with a post hoc Tukey test a,b). SFA, saturated fatty acids; MUFA, monounsaturated fatty acids; PUFA, polyunsaturated fatty acids; n.d., not detected.

Regarding the extraction methods, the fatty acid profile and relative abundance of the lipid extracts were very similar, irrespective of the approach employed. Only minor differences were found in certain fatty acids, but the overall impact of the extraction method was negligible. These results align with previous studies that reported no substantial changes in the fatty acid composition, independent of the method or solvent used.^11,42^ These outcomes open new possibilities for using alternative techniques to extract microalgal lipids, such as ultrasound, with the potential to employ greener solvents.

### 3.2 Overview of Oxylipins Profile in Different Microalgae Species

**Figure 1:**
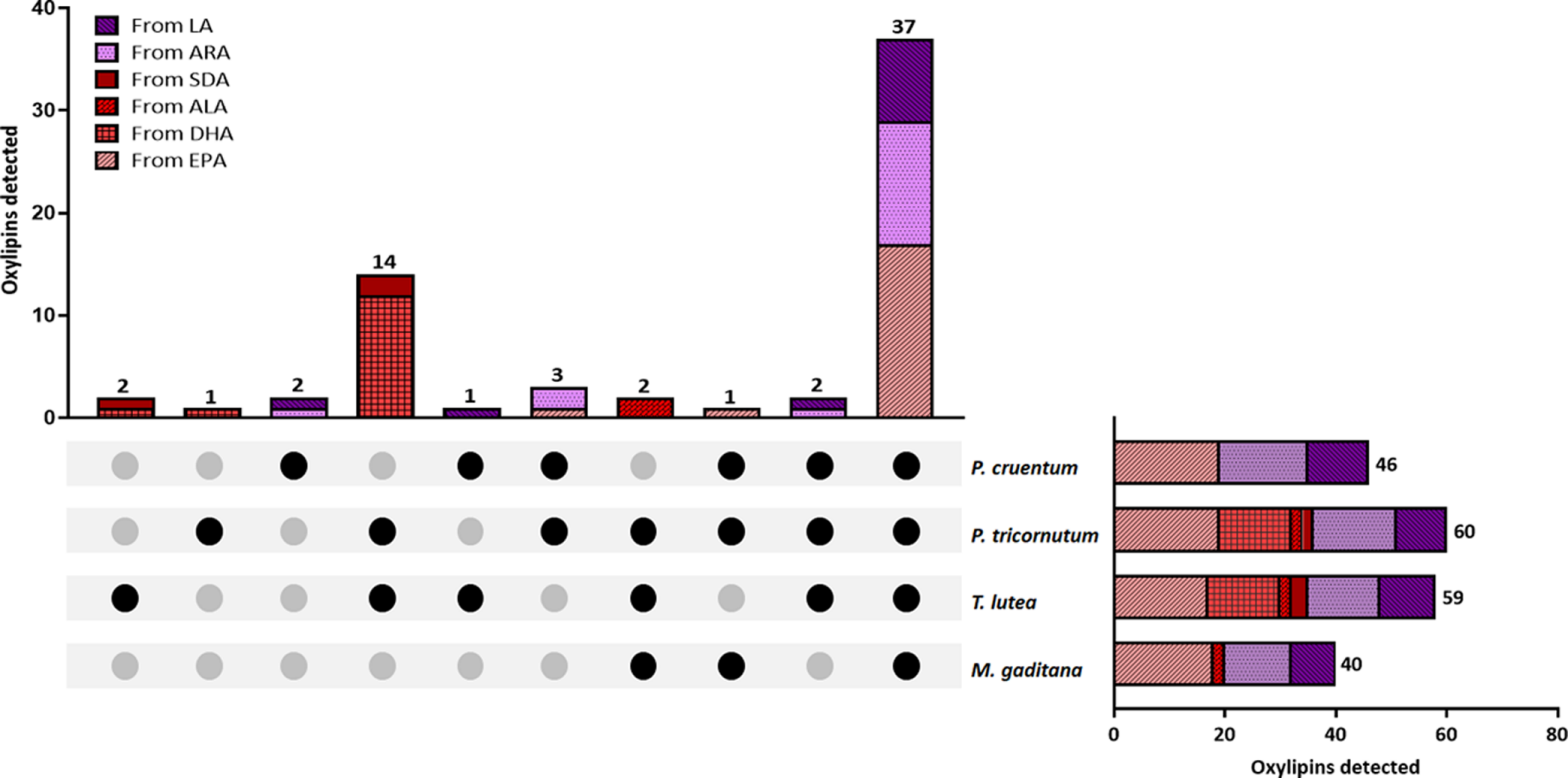
Overview of the number of identified oxylipins in different microalgae species: *M. gaditana*, *T. lutea*, *P. tricornutum*, and *P. cruentum*. The top graph shows the number of common oxylipins between the species marked with black circles. The right graph shows the total number of oxylipins for each species. Different colors and patterns in both bar charts represent the precursor fatty acid of the oxylipins. EPA: eicosapentaenoic acid; DHA: docosahexaenoic acid; ALA: linolenic acid; SDA: stearidonic acid; ARA: arachidonic acid; LA: linoleic acid.

In light of the high PUFA content and the specific fatty acid compositions, our strategy involved targeting specific oxylipins of interest derived from free n-3 (ALA, SDA, EPA, and DHA) and n-6 (LA and ARA) fatty acids, as the nonesterified version of oxylipins is considered to be the biologically relevant one.^19^Figure 1 shows an overview of the number of identified oxylipins in the different microalgae species investigated. A total of 68 oxylipins were detected, with 37 being common across all microalgae species and 5 being exclusive to individual species. In pairwise comparisons, *T. lutea* and *P. tricornutum* were found to share the highest number of oxylipins (16), specifically those derived from DHA, ALA, and SDA. In terms of the total number of identified oxylipins, the species were ranked as *P. tricornutum* (60) > *T. lutea* (59) > *P. cruentum* (46) > *M. gaditana* (40). Therefore, these microalgae were confirmed to be a potential oxylipin reservoir, as we hypothesized.

In general, the oxylipin pattern aligns with the fatty acid profile described for these microalgal lipid extracts, with few exceptions. For example, in *P. tricornutum*, oxylipins derived from ALA and SDA were identified, even though the precursor fatty acid was not detected; however, the concentration of these oxylipins was low (see Section 3.4 for more detailed information). Similarly, in *T. lutea* extracts, oxylipins derived from EPA and ARA were identified, but not the precursor fatty acid (see Sections 3.4 and 3.5 for more detailed information). As already reported by other authors, the abundance of PUFA substrates surprisingly differs from their utilization in oxylipin biosynthetic pathways. For example, macroalgae belonging to the Ochrophyta phylum are relatively poor in C18 PUFA; however, they commonly utilize this substrate in LOX-initiated biosynthetic pathways.^43^ Nevertheless, the information available on microalgal oxylipin biosynthesis remains scarce, and other unknown oxylipin biosynthetic pathways might be at play, as hypothesized in Sections 3.4. and 3.5.

### 3.3 Impact of Extraction Method on Oxylipins Profile

**Figure 2:**
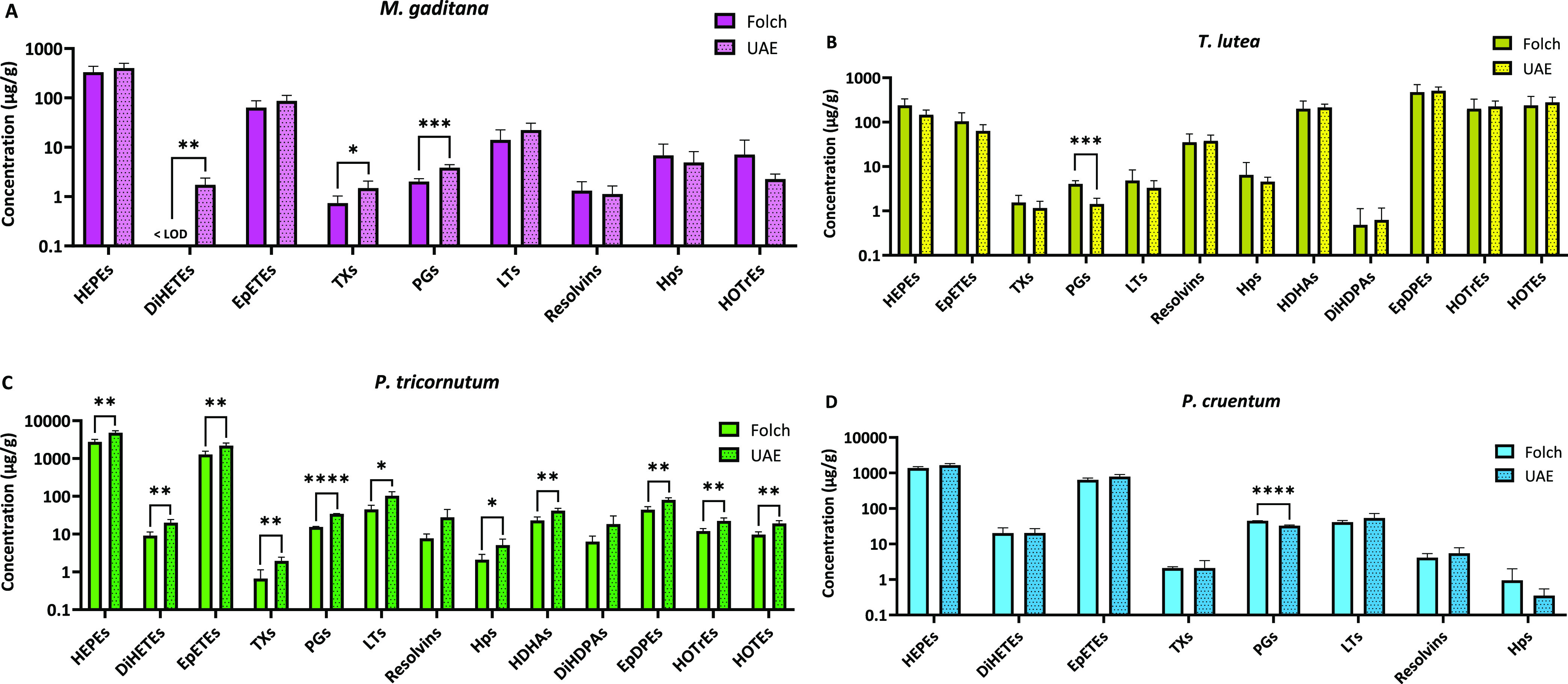
Impact of extraction method on omega-3-derived oxylipins from (A) *M. gaditana*, (B) *T. lutea*, (C) *P. tricornutum*, and (D) *P. cruentum*. Data is shown as mean ± SD (*n* ≥ 3) of the sum of each oxylipin class. Statistically significant difference between Folch and UAE for each group is expressed as * (*p* < 0.05), **(*p* < 0.01), *** (*p* < 0.001), and **** (*p* < 0.0001), as determined by unpaired *t* test. HEPEs: hydroxyeicosapentaenoic acids; DiHETEs: dihydroxyeicosatetraenoic acids; EpETE: epoxyeicosatetraenoic acids; TXs: thromboxanes; PGs: prostaglandins; LTs: leukotrienes; Hps: hydroperoxides; HDHAs: hydroxydocosahexaenoic acids; DiHDPAs: dihydroxydocosapentaenoic acids; EpDPEs: epoxydocosapentanoic acids; HOTrEs: hydroxyoctadecatrienoic acids; HOTEs: hydroxyoctadecatetraenoic acids; LOD: limit of detection.

**Figure 3:**
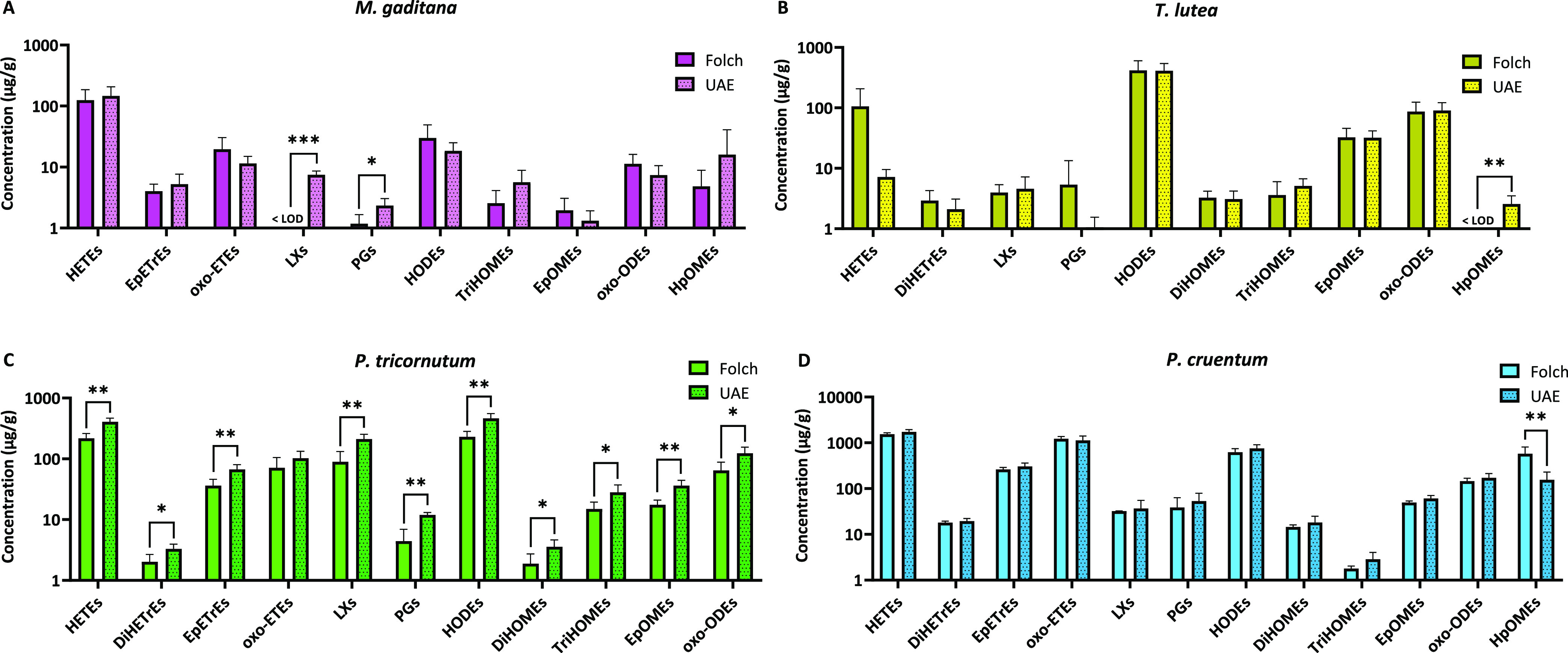
Impact of extraction method on omega-6-derived oxylipins from (A) *M. gaditana*, (B) *T. lutea*, (C) *P. tricornutum*, and (D) *P. cruentum*. Data is shown as mean ± SD (*n* ≥ 3) of the sum of each oxylipin class. Statistically significant difference between Folch and UAE for each group is expressed as * (*p* < 0.05), **(*p* < 0.01), and *** (*p* < 0.001), as determined by unpaired *t* test. HETEs: hydroxyeicosatetraenoic acids; DiHETrEs: dihydroxyeicosatrienoic acids; EpETrEs: epoxyeicosatrienoic acids; Oxo-ETEs: oxoeicosatetraenoic acids; LXs: lipoxins; PGs: prostaglandins; HODEs: hydroxyoctadecadienoic acids; DiHOMEs: dihydroxyoctadecenoic acids; TriHOMEs: trihydroxyoctadecenoic acids; EpOMEs: epoxyoctadecenoic acids; Oxo-ODEs: oxooctadecadienoic acids; HpOMEs: hydroperoxyoctadecenoic acids; LOD: limit of detection.

To the best of our knowledge, this is the first time that the impact of the extraction method on the microalgal oxylipin profile has been reported. Figures 2 and 3 show the impact of the extraction method on n-3- and n-6-derived oxylipin concentration, grouped by oxylipin class, across the four microalgal extracts. Overall, with a few exceptions detailed below, the ultrasound approach yields comparable concentrations of n-3- and n-6-derived oxylipins to those of the Folch method, suggesting it as an alternative to conventional approaches. While an elevated concentration of oxylipins might be initially perceived as a negative outcome, it is crucial to emphasize that, as discussed below, most of them play essential roles in various biological functions and have positive implications for the human body.^44^

For *M. gaditana* (Figure 2A), the ultrasound approach yielded slightly higher concentrations of a few n-3-derived oxylipin classes, including dihydroxyeicosatetraenoic acids (DiHETEs), thromboxanes (TXs), and prostaglandins (PGs). Similarly, for *P. tricornutum* (Figure 2C), the ultrasound method also resulted in slightly higher concentrations compared to those of the conventional Folch method, observed across almost all oxylipin classes (excluding resolvins and dihydroxydocosapentaenoic acids). In the case of *T. lutea* and *P. cruentum* (Figure 2B,D), comparable results were obtained for both methods, showing only a slight reduction in PGs for UAE compared with the conventional Folch method.

Regarding the oxylipin profile derived from n-6 fatty acids, a similar pattern was observed. For instance, in *M. gaditana* (Figure 3A), the ultrasound approach resulted in moderately higher concentrations of lipoxins (LXs) and PGs, whereas for *T. lutea* and *P. cruentum* (Figure 3B,D), only the concentration of hydroperoxyoctadecenoic acids (HpOMEs) was affected. Similarly, a slight positive effect of the ultrasound approach on n-6-derived oxylipins was noted for *P. tricornutum* (Figure 3C), where significantly higher concentrations of almost all oxylipin classes were observed. Thus, these results underscore the potential application of alternative extraction methods to recover these specific bioactive compounds from different microalgae species.

### 3.4 Omega-3-Derived Oxylipins from Microalgae

As the use of ultrasound proved to be a reliable method for extracting oxylipins, the characterization of the oxylipin profile from the different microalga species and their quantification were based on the UAE results (Table 2), although results from Folch are also available in Tables S2 and S3.

**Table 2:** Omega-3-Derived Oxylipins from *M. gaditana*, *T. lutea*, *P. tricornutum*, and *P. cruentum* after Ultrasound-Assisted Extraction^a^

		concentration (μg/g)			
precursor fatty acid	free oxylipin	*M. gaditana*	*T. lutea*	*P. tricornutum*	*P. cruentum*
EPA	5-HEPE	186.7 ± 86.1^a^	4.2 ± 2.2^b^	178.9 ± 55.3^a^	37.9 ± 9.6^b^
	8-HEPE	21.3 ± 8.9^c^	18.1 ± 9.6^c^	552.7 ± 157.6^a^	182.3 ± 40.0^b^
	12-HEPE	6.5 ± 2.7^c^	8.6 ± 4.5^c^	248.1 ± 74.2^a^	86.5 ± 19.6^b^
	15-HEPE	16.7 ± 6.4^c^	13.9 ± 7.0^c^	384.0 ± 111.7^a^	148.4 ± 35.8^b^
	8,9-EpETE	0.2 ± 0.1^c^	0.2 ± 0.1^c^	5.4 ± 1.5^a^	1.8 ± 0.5^b^
	11,12-EpETE	6.2 ± 2.2^c^	6.2 ± 3.2^c^	167.7 ± 48.7^a^	57.8 ± 12.1^b^
	14,15-EpETE	21.4 ± 9.4^c^	16.9 ± 9.2^c^	531.5 ± 136.5^a^	216.9 ± 44.6^b^
	17,18-EpETE	58.9 ± 23.6^c^	39.9 ± 22.5^c^	1479.4 ± 356.4^a^	515.2 ± 99.6^b^
	11-HEPE	28.8 ± 13.1^c^	21.6 ± 11.6^c^	665.1 ± 189.4^a^	246.9 ± 54.6^b^
	9-HEPE	55.7 ± 29.2^bc^	18.8 ± 10.3^c^	508.5 ± 147.5^a^	164.0 ± 48.5^b^
	5,6-DiHETE	1.7 ± 0.6^b^	<LOD	17.7 ± 3.9^a^	1.9 ± 0.4^b^
	14,15-DiHETE	<LOD	<LOD	1.6 ± 0.9^b^	17.7 ± 6.3^a^
	TXB_3_	1.3 ± 0.5^a^	1.1 ± 0.4^a^	1.8 ± 0.5^a^	1.9 ± 1.2^a^
	PGF_3α_	0.1 ± 0.0^b^	0.2 ± 0.1^b^	0.9 ± 0.2^ab^	1.8 ± 0.9^a^
	PGE_3_	3.8 ± 1.2^b^	1.3 ± 0.7^b^	33.6 ± 7.7^a^	31.1 ± 10.6^a^
	Resolvin E_1_	1.2 ± 0.6^b^	1.5 ± 1.2^b^	22.7 ± 18.5^a^	6.0 ± 2.5^b^
	LTB_5_	22.2 ± 8.6^c^	3.3 ± 1.5^c^	103.1 ± 30.0^a^	53.7 ± 18.8^b^
	18-HEPE	93.8 ± 37.7^c^	63.6 ± 35.9^c^	2356.5 ± 567.7^a^	820.7 ± 158.6^b^
	12/15-HpEPE	5.3 ± 3.5^a^	0.1 ± 0.0^b^	0.2 ± 0.1^b^	0.4 ± 0.2^b^
DHA	17-HDHA	<LOD	93.3 ± 30.9^a^	18.2 ± 4.9^b^	<LOD
	7,8-EpDPE	<LOD	19.1 ± 6.9^a^	2.8 ± 1.0^b^	<LOD
	10,11-EpDPE	<LOD	29.8 ± 10.5^a^	5.4 ± 2.1^b^	<LOD
	13,14-EpDPE	<LOD	81.9 ± 31.9^a^	14.7 ± 3.9^b^	<LOD
	16,17-EpDPE	<LOD	137.2 ± 55.4^a^	23.5 ± 4.9^b^	<LOD
	19,20-EpDPE	<LOD	201.7 ± 77.9^a^	28.4 ± 7.2^b^	<LOD
	16,17-DiHDPA	<LOD	0.6 ± 0.5	<LOD	<LOD
	19,20-DiHDPA	<LOD	<LOD	<LOD	<LOD
	7-HDHA	<LOD	40.4 ± 15.0^a^	7.4 ± 1.9^b^	<LOD
	Resolvin D_1_	<LOD	9.2 ± 4.3^a^	2.5 ± 0.4^b^	<LOD
	Resolvin D_2_	<LOD	27.7 ± 12.9^a^	4.6 ± 1.2^b^	<LOD
	11-HDHA	<LOD	30.2 ± 10.8^a^	5.5 ± 2.3^b^	<LOD
	14-HDHA	<LOD	37.0 ± 13.5^a^	7.7 ± 2.8^b^	<LOD
	17-HpDHA	<LOD	4.4 ± 1.2^a^	4.9 ± 2.3^a^	<LOD
	7,17-DiHDPA	<LOD	<LOD	16.4 ± 10.7	<LOD
ALA	9-HOTrE	0.8 ± 0.6^c^	66.3 ± 24.8^a^	8.8 ± 2.3^b^	<LOD
	13-HOTrE	1.7 ± 0.2^c^	182.7 ± 73.9^a^	15.7 ± 4.4^b^	<LOD
SDA	6-HOTE	<LOD	170.2 ± 60.5^a^	14.3 ± 4.0^b^	<LOD
	9-HOTE	<LOD	4.3 ± 1.9	<LOQ	<LOD
	13-HOTE	<LOD	133.9 ± 74.9^a^	6.8 ± 1.3^b^	<LOD

a Data is shown as mean ± SD (*n* ≥ 3). Different lowercase letters (a, b, c) show statistically significant differences (*p* < 0.05). EPA: eicosapentaenoic acid; DHA: docosahexaenoic acid; ALA: linolenic acid; SDA: stearidonic acid; HEPE: hydroxyeicosapentaenoic acid; EpETE: epoxyeicosatetraenoic acid; DiHETE: dihydroxyeicosatetraenoic acid; TX: thromboxane; PG: prostaglandin; LT: leukotriene; HpEPE: hydroperoxyeicosapentaenoic acid; HDHA: hydroxydocosahexaenoic acid; EpDPE: epoxydocosapentanoic acid; DiHDPA: dihydroxydocosapentaenoic acid; HpDHA: hydroperoxydocosahexaenoic acid; HOTrE: hydroxyoctadecatrienoic acid; HOTE: hydroxyoctadecatetraenoic acid; LOD: limit of detection; LOQ: limit of quantification.

As shown in Table 2, *M. gaditana* extracts contained several EPA-derived oxylipins including hydroxide, epoxide, dihydroxide, and hydroperoxide derivatives as well as two prostaglandins, a thromboxane, a resolvin, and a leukotriene. From the aforementioned classes, hydroxyeicosapentaenoic acid (HEPE) derivatives generally stood out for their high yields in *M. gaditana* extracts, with concentrations between 6.5 ± 2.7 μg/g for 12-HEPE and 186.7 ± 86.1 μg/g for 5-HEPE. Other prominent oxylipins from this fatty acid in *M. gaditana* were the epoxyeicosatetraenoic acid (EpETE) derivatives, especially 17,18-EpETE, with a content of 58.9 ± 23.6 μg/g, and LTB_5_, with a concentration of 22.2 ± 8.6 μg/g. As mentioned in the introduction, to our knowledge, only 15-HEPE had been previously identified in *M. gaditana*. Despite this, 15-HEPE did not show the highest concentration (16.7 ± 6.4 μg/g) among the EPA-derived oxylipins detected. Consequently, this study provided the most extensive oxylipin characterization of this microalgae to date, broadening the potential applications of this species.

The same EPA-derived oxylipins were also present in the other three microalgae species, except for 5,6-DiHETE and 14,15-DiHETE, which were not identified in *T. lutea*. Nonetheless, a considerable number of EPA-derived oxylipins were still detected in this species, despite EPA not being present in its fatty acid composition (Table 1). This was observed to a significantly lower extent than *P. tricornutum* and *P. cruentum* for almost all cases, with the majority of them showing average concentrations below 10 μg/g. However, some HEPE and EpETE oxylipins were present in *T. lutea* in amounts comparable to those of *M. gaditana*, with 18-HEPE and 17,18-EpETE as the most abundant, displaying concentrations of 63.6 ± 35.9 and 39.9 ± 22.5 μg/g, respectively. While the fatty acid composition greatly depends on the microalgae growing conditions, there is existing evidence that *T. lutea* is able to generate small amounts of EPA under certain situations.^45^ Our results, therefore, suggest the possible presence of highly efficient lipoxygenases and cyclooxygenases in this species, capable of reducing the low EPA content of this microalgae to nondetectable amounts. Nevertheless, this hypothesis must be confirmed by future studies. In addition, growing conditions can modify the conversion between fatty acids by promoting or inhibiting the activity of desaturases and elongases in microalgae such as *M. gaditana* and *P. tricornutum*.^46^ While no evidence of this has been shown so far for *T. lutea*, it is likely that the role of the growing conditions on these enzymes may have contributed to the results obtained.

From a qualitative perspective, *P. tricornutum* and *P. cruentum* shared a similar EPA-derived oxylipin profile. Furthermore, HEPE and EpETE were the most representative oxylipin groups in both species. Nevertheless, *P. tricornutum* lipid extracts yielded a significantly higher content of all oxylipins belonging to these classes, reaching remarkably high values in specific oxylipins such as 18-HEPE and 17,18-EpETE (2356.5 ± 567.7 and 1479.4 ± 356.4 μg/g, respectively). On the other hand, this trend was not observed for all EPA-derived oxylipin classes, as there were instances where no significant difference was found between these two microalgae. This was the case for EPA-derived prostaglandins, thromboxanes, and hydroperoxides. Interestingly, unlike the other classes, DiHETE derivatives showed a different trend depending on the specific positional isomer with 5,6-DiHETE displaying a significantly higher concentration in *P. tricornutum* and 14,15-DiHETE being more prominent in *P. cruentum*. Furthermore, the concentration of 5,6-DiHETE in *P. tricornutum* was similar to the concentration of 14,15-DiHETE in *P. cruentum* (17.7 ± 3.9 and 17.7 ± 6.3 μg/g, respectively), whereas the amount of 5,6-DiHETE in *P. cruentum* (1.9 ± 0.4 μg/g) was comparable to that of 14,15-DiHETE in *P. tricornutum* (1.6 ± 0.9 μg/g). Therefore, these findings might hint toward a species-dependent difference in the enzymatic systems responsible for the production of this oxylipin class.

A series of DHA-derived oxylipins were also found within the lipid extracts of *T. lutea* and *P. tricornutum*. *T. lutea* generally showed a significantly higher concentration of this group compared to *P. tricornutum*, possibly because DHA is not one of the most predominant fatty acids in *P. tricornutum*, contrary to that of *T. lutea*. This was the case for all DHA-derived oxylipins except 7,17-dihydroxydocosapentaenoic acid, which was detected only in *P. tricornutum* extracts. Overall, the most prominent DHA-derived oxylipins in terms of concentration for both microalgae were the epoxydocosapentaenoic acid (EpDPE) derivatives, among which 19,20-EpDPE exhibited the highest mean content in both microalgae (201.7 and 28.4 μg/g for *T. lutea* and *P. tricornutum*, respectively). In addition, 17-hydroxydocosahexaenoic acid stood out as another main DHA-derived oxylipin, reaching concentrations of 93.3 ± 30.9 μg/g for *T. lutea* and 18.2 ± 4.9 μg/g for *P. tricornutum*. Likewise, resolvins D_1_ and D_2_ were found in *T. lutea* in a significantly higher concentration than in *P. tricornutum*, while a trace amount of 16,17-dihydroxydocosapentaenoic acid could only be detected in *T. lutea*. However, despite all of the described differences, the content of 17-hydroperoxydocosahexaenoic acid was comparable in both species.

ALA- and SDA-derived oxylipins remain a much less studied group, likely due to the preferred interest for long-chain PUFA derivatives over C18 fatty acids. However, a few oxylipin structures have been described for them.^47^ In the present study, two ALA-derived hydroxyoctadecatrienoic acid (HOTrE) oxylipins (9-HOTrE and 13-HOTrE) were detected in lipid extracts obtained from *M. gaditana*, *T. lutea*, and *P. tricornutum*. Nevertheless, the concentration of these ALA-derived oxylipins was statistically significantly higher in *T. lutea* than in the other two species, reaching a concentration of 182.7 ± 73.9 μg/g in the case of 13-HOTrE. Their concentration was especially low in *M. gaditana* (<2 μg/g), with *P. tricornutum* showing significantly higher values (8.8 ± 2.3 μg/g for 9-HOTrE and 15.7 ± 4.4 μg/g for 13-HOTrE), but still clearly lower than *T. lutea*. Considering that ALA represents a relevant lipid within the fatty acid composition of *T. lutea* (Table 1), the significantly higher content of ALA-derived oxylipins in *T. lutea* was expected. In contrast, *P. tricornutum* and *M. gaditana* did not show detectable ALA amounts by GC-MS, which suggests the possibility of low quantities of ALA being synthesized in *M. gaditana* and *P. tricornutum* and quickly converted by lipoxygenases or the existence of oxylipin conversion pathways that have not yet been described.

A similar outlook was observed for SDA-derived oxylipins, where three hydroxyoctatetraenoic acid (HOTE) derivatives were detected in *T. lutea* and *P. tricornutum*, with a significantly higher concentration in *T. lutea*. Among them, 6-HOTE and 13-HOTE represented the main oxylipins from this group, with concentrations in *T. lutea* of 170.2 ± 60.5 and 133.9 ± 74.9 μg/g, respectively. Meanwhile, a modest amount of 9-HOTE (4.3 ± 1.9 μg/g) could be determined in *T. lutea* extracts, while in *P. tricornutum* only a nonquantifiable amount could be detected. These results point toward the existence, for these microalgae species, of a certain positional directionality in the production of SDA-derived oxylipins, when grown under specific conditions.

In general, although the relative percentage of n-3 PUFAs in *P. tricornutum* and *P. cruentum* extracts was lower than that in *M. gaditana* (Table 1), a higher content of n-3 PUFA oxylipins was found in these two microalgae. This could indicate that the enzyme system responsible for oxylipin biosynthesis in these species might manifest higher activity and efficiency than in *M. gaditana*. In this sense, numerous studies involving oxylipin determination in diatoms and red algae have repeatedly reported them to show LOX activity, supporting our explanation, as evidence on this remains scarcer about other algae from the Ocraphyta phylum including *M. gaditana* and *T. lutea*.^15^ Nevertheless, the amount of n-3-derived oxylipins determined in *T. lutea* should not be neglected, as the high n-3/n-6 fatty acid ratio in this species clearly led to the formation of a remarkably large set of different n-3-derived oxylipins, with those formed from DHA, ALA, and SDA generally showing the highest concentrations of the four species studied.

Regarding the chemical nature of the analyzed n-3 PUFA oxylipins overall, the presence of a few hydroperoxide derivatives in small amounts compared to other oxylipin classes stood out (Figures S1–S4). This can be attributed to the fact that hydroperoxides are the main intermediaries in the LOX oxylipin pathway, quickly being converted into other more stable oxylipin classes.^17^ Therefore, the presence of more hydroperoxide chemical species in the oxylipin biosynthetic pathway needs to be assumed (Figures S1–S6).

Interestingly, the four microalgae displayed certain differences in their oxylipin profile within each n-3 fatty acid. In this context, higher contents of specific individual oxylipins, especially within the same oxylipin class, point toward a selectivity or directionality of the pathways depending on the microalgae species. For instance, 5-HEPE was the most prominent EPA-derived hydroxide in *M. gaditana*. At the same time, monohydroxy oxylipins with the hydroxy group in a higher position number were more abundant in *P. tricornutum* and *P. cruentum*. This suggests a higher activity of a 5-LOX in *M. gaditana* and a more prominent role of higher position number LOXs in the other two species. This is further supported by the fact that the content of hydroperoxyeicosapentaenoic acids (HpEPEs), 12-HpEPE and 15-HpEPE, was significantly higher in *M. gaditana* (5.3 ± 3.5 μg/g combined) while only trace amounts (<0.5 μg/g) could be found for the other species, indicating a less relevant role of enzymes such as 15-LOX in *M. gaditana*.

Omega-3-derived oxylipins have increasingly received attention in recent years due to the growing evidence regarding their biological effects.^48^ For instance, oxylipins derived from EPA, DHA, and ALA are considered potent anti-inflammatory compounds.^44^ However, the biological activity of other n-3-derived oxylipins has not yet been assessed, such as SDA-derived oxylipins. Previous studies on animal or cell models have associated individual n-3-derived oxylipins with specific biological effects, including antitumorigenic activities and effects on glucose and lipid metabolism and human platelet aggregation.^49^ Given that microalgae serve as a valuable source of n-3 fatty acids, it is reasonable to expect that interest in their oxylipins would also rise. Microalgae are considered a sustainable source of n-3 and n-6 biomolecules compared to fish and plants, respectively, due to their rapid growth, high biomass yield, and potential scalability of their production.^50,51^ As highlighted by Gabbs et al., different oxylipin classes, as well as differently positioned chemical groups within the same class, can have an impact on their biological activity.^18^ Hence, exploring different microalgae could potentially open the path to developing diverse applications from their extracts. However, further comprehensive research is necessary to assess this potential.

### 3.5 Omega-6-Derived Oxylipins from Microalgae

In relation to n-6-derived oxylipins, attention was paid to those derived from ARA and LA (Table 3). While microalgae have emerged as a valuable source of ARA, considerations in terms of ARA-derived oxylipins have been only pointed out in a limited number of microalgae species such as *P. cruentum*,^52^ despite n-6-derived oxylipins having shown in certain occasions higher biological potency than the n-3-derived counterpart.^53^ In this study, a series of ARA-derived oxylipins were determined, including hydroxyeicosatetraenoic (HETE), oxoeicosatetraenoic (oxo-ETE), epoxyeicosatrienoic (EpETrE), and dihydroxyeicosatrienoic (DiHETrE) derivatives as well as lipoxin A_4_ and PGE_2_. Most of these oxylipins were determined in the lipid extracts of *M. gaditana*, *P. tricornutum*, and *P. cruentum*, whereas only a few of them, in low amounts (<0.5 μg/g), were detected in *T. lutea*.

**Table 3:** Omega-6-Derived Oxylipins from *M. gaditana*, *T. lutea*, *P. tricornutum*, and *P. cruentum* after Ultrasound-Assisted Extraction^a^

		concentration (μg/g)			
precursor fatty acid	free oxylipin	*M. gaditana*	*T. lutea*	*P. tricornutum*	*P. cruentum*
ARA	5-HETE	124.9 ± 60.8^b^	1.4 ± 0.8^c^	53.2 ± 15.7^bc^	228.5 ± 70.1^a^
	8-HETE	3.6 ± 2.4^bc^	0.9 ± 0.6^c^	39.6 ± 12.4^b^	142.8 ± 36.7^a^
	9-HETE/11-HETE	3.9 ± 2.8^b^	0.8 ± 0.5^b^	41.9 ± 14.4^b^	224.5 ± 61.6^a^
	12-HETE	10.8 ± 6.7^b^	3.0 ± 1.9^b^	147.6 ± 44.2^b^	583.5 ± 160.6^a^
	15-HETE	4.5 ± 3.1^b^	1.3 ± 0.9^b^	65.5 ± 16.3^b^	255.8 ± 64.2^a^
	20-HETE	<LOD	<LOD	62.4 ± 26.3^b^	311.8 ± 80.0^a^
	5-oxo-ETE	6.0 ± 1.5^b^	<LOD	39.3 ± 19.8^b^	687.9 ± 240.5^a^
	15-oxo-ETE	4.6 ± 3.0^b^	<LOD	55.9 ± 21.1^b^	367.4 ± 89.6^a^
	5,6-EpETrE	0.2 ± 0.1^b^	<LOD	0.6 ± 0.2^b^	2.0 ± 0.6^a^
	8,9-EpETrE/11,12-EpETrE	2.5 ± 1.7^b^	<LOD	26.0 ± 9.0^b^	141.0 ± 38.7^a^
	14,15-EpETrE	2.6 ± 1.7^b^	<LOD	40.1 ± 9.2^b^	159.9 ± 40.5^a^
	5,6-DiHETrE	<LOD	<LOD	1.0 ± 0.2^b^	4.4 ± 1.1^a^
	11,12-DiHETrE	<LOD	<LOD	<LOD	1.6 ± 0.9
	14,15-DiHETrE	<LOD	2.0 ± 0.9^b^	2.2 ± 0.6^b^	12.5 ± 2.1^a^
	LXA_4_	7.1 ± 1.1^b^	4.3 ± 2.5^b^	199.9 ± 40.0^a^	34.7 ± 17.5^b^
	PGE_2_	2.3 ± 0.7^bc^	1.1 ± 0.5^c^	11.9 ± 1.1^a^	53.0 ± 26.0^ab^
LA	12,13-EpOME	0.8 ± 0.5^c^	20.4 ± 8.6^b^	22.0 ± 6.6^b^	38.7 ± 9.1^a^
	9,10-EpOME	0.6 ± 0.4^c^	14.2 ± 5.6^b^	16.9 ± 5.5^ab^	26.1 ± 6.3^a^
	9,10-DiHOME	<LOD	1.4 ± 0.7^b^	<LOD	10.4 ± 6.6^a^
	12,13-DiHOME	<LOD	1.8 ± 0.9^b^	3.7 ± 1.1^b^	8.3 ± 1.7^a^
	9-HODE	5.0 ± 2.1^c^	111.1 ± 44.2^b^	124.8 ± 36.7^ab^	186.0 ± 49.2^a^
	13-HODE	15.2 ± 6.9^c^	336.1 ± 135.5^b^	380.7 ± 93.9^b^	639.8 ± 160.0^a^
	9-oxo-ODE	7.5 ± 3.2^c^	69.7 ± 30.1^b^	111.2 ± 31.8^ab^	152.0 ± 41.1^a^
	13-oxo-ODE	<LOD	21.9 ± 10.1^a^	13.6 ± 5.0^a^	21.7 ± 5.5^a^
	9,10,13-TriHOME	5.5 ± 3.1^b^	4.0 ± 1.5^b^	27.6 ± 9.0^a^	2.8 ± 1.1^b^
	9,12,13-TriHOME	<LOD	1.0 ± 0.2	<LOD	<LOQ
	Total HpOME	18.3 ± 28.6^b^	2.9 ± 1.1^b^	<LOD	178.4 ± 85.4^a^
	13-HpODE	0.6 ± 0.7^b^	0.1 ± 0.0^b^	<LOD	5.4 ± 2.8^a^
	9-HpOME	<LOD	<LOD	<LOD	0.3 ± 0.2

a Data is shown as mean ± SD (*n* ≥ 3). Different lowercase letters (a, b, c) show statistically significant differences. ARA: arachidonic acid; LA: linoleic acid; HETE: hydroxyeicosatetraenoic acid; Oxo-ETE: oxoeicosatetraenoic acid; EpETrE: epoxyeicosatrienoic acid; DiHETrE: dihydroxyeicosatrienoic acid; LX: lipoxin; PG: prostaglandin; EpOME: epoxyoctadecenoic acid; HOME: hydroxyoctadecenoic acid; HODE: hydroxyoctadecadienoic acid; Oxo-ODE: oxooctadecadienoic acid; HpOME: hydroperoxyoctadecenoic acid; HpODE: hydroperoxyoctadecadienoic acid; LOD: limit of detection; LOQ: limit of quantification.

Interestingly, no dihydroxy oxylipins were identified in *M. gaditana*, and 5-HETE was the main oxylipin found in this species (124.9 ± 60.8 μg/g), once again suggesting the presence of a highly effective 5-LOX. In contrast, the rest of ARA-derived oxylipins showed modest amounts in *M. gaditana* (<20 μg/g). In the case of *P. tricornutum*, most of its ARA-derived oxylipins displayed a content comparable to that of *M. gaditana* extracts. Apart from 5,6-DiHETrE and 14,15-DiHETrE, which were not detected in the latter, only LXA_4_ and PGE_2_ exhibited a statistically significantly higher concentration in *P. tricornutum* (199.9 ± 40.0 and 11.9 ± 1.1 μg/g) compared to *M. gaditana*. Nevertheless, the oxylipin distribution within the same class was not identical for both species, as 12-HETE was the main HETE for *P. tricornutum* (147.6 ± 44.2 μg/g) as opposed to 5-HETE in *M. gaditana*, which hints at the different uses of oxylipin pathways between them. On the other hand, *P. cruentum* extracts showed the highest content of almost each of the studied ARA-derived oxylipins, which can be explained by their richness in this fatty acid (22.7 ± < 0.1%). Among them, HETE and oxo-ETE derivatives showed the highest concentrations (>100 μg/g), with the content of 12-HETE and 5-oxo-ETE especially pronounced (583.5 ± 160.6 μg/g and 687.9 ± 240.5 μg/g, respectively). However, out of all four species, the highest content of LXA_4_ was found in *P. tricornutum*, which may point toward a specific better efficiency in synthesizing this oxylipin by this microalgae species. In this sense, PGE_2_ has been stated to induce the formation of LXA_4_ in human cells.^54^ However, it is currently unclear whether this phenomenon occurs in microalgae, as well.

Additionally, LA-derived oxylipins, including HpOME, epoxyoctadecenoic (EpOME), oxooctadecadienoic (oxo-ODE), hydroxyoctadecadienoic (HODE), dihydroxyoctadecenoic (DiHOME), and trihydroxyoctadecenoic acid (TriHOME) derivatives, were determined in the microalgae species with significant differences among them. Once again, *P. cruentum* generally showed the highest content of these oxylipins, as expected due to its higher relative percentage in LA in comparison to the other three species (Table 1). Among them, 9-HODE, 13-HODE, and 9-oxo-ODE were particularly prominent, with concentrations of 186.0 ± 49.2, 639.8 ± 160.0, and 152.0 ± 41.1 μg/g, respectively. In contrast, no significant difference was found for 13-oxo-ODE between *P. cruentum*, *T. lutea*, and *P. tricornutum*, further evidencing the different behavior of positional isomeric oxylipins in microalgae. On the other hand, 13-oxo-ODE was not detected in *M. gaditana*, which also stood out for exhibiting the significantly lowest amount of 9-oxo-ODE, HODEs, and EpOMEs, as well as a lack of the analyzed DiHOME derivatives. Interestingly, TriHOME derivatives showed a different trend than the rest of LA-derived oxylipins, as *P. tricornutum* possessed the highest 9,10,13-TriHOME content (27.6 ± 9.0 μg/g) and *T. lutea* proved to be the only species where 9,12,13-TriHOME could be quantified (1.0 ± 0.2 μg/g). Surprisingly, no hydroperoxide derivatives were found in the lipid extracts of *P. tricornutum*, which may be linked to a highly efficient enzymatic system that depletes the content of short-lived intermediary molecules such as hydroperoxides, in favor of other stable oxylipin groups.^17^

Interestingly, previous studies on diatoms have suggested the complete absence of C18 PUFA LOX-derived products.^17,55^ This contrasts with our data on *P. tricornutum*, for which several LOX oxylipins derived from SDA, ALA, and LA were identified. Diatoms, however, consist of a large group of microalgae genera,^56^ which may explain that additional enzymatic activities are present in species that were not studied in this regard until now, such as *P. tricornutum*. In addition, there is prior evidence that the genome of *P. tricornutum* does not code for obvious prostaglandin-generating enzymes.^30^ Therefore, the presence of alternative unknown biosynthetic pathways in microalgae cannot be ruled out.

As opposed to n-3-derived oxylipins, which have been generally associated with anti-inflammatory effects by prior research, n-6-derived oxylipins have been claimed to be responsible for both pro- and anti-inflammatory roles depending on their specific structure, with even some of them being able to induce contradictory effects in a situational-specific manner.^20,57^ This adds another layer of complexity to the potential use of microalgal oxylipins, highlighting the need to evaluate the biological activity of extracts of each species.

In summary, our findings proved, for the first time, that the ultrasound-assisted extraction method represents a green alternative that can successfully extract microalgal fatty acids and their oxylipins simultaneously, potentially opening new possibilities for the application of microalgae extracts. To our knowledge, the present study provided the most extensive characterization to date of the oxylipin profile of these four microalgae species, identifying a total of 68 free oxylipins derived from n-3 and n-6 fatty acids. Considering the great variety of oxylipin classes in the four microalgae of study and how they differently contribute to the total oxylipin profile, elucidating the biological effects of their lipid extracts can present a challenge. Consequently, future research should focus on evaluating the biological activity of these extracts, preferably using green extraction methods, such as the one developed in our study.

## Supporting Information Available

Postulated oxylipin biosynthetic pathways in microalgae (Figures S1–S6); additional experimental details for LC-MS/MS analysis (Table S1); and omega-3 and omega-6 oxylipin determination in microalgal Folch extracts (Tables S2 and S3) (PDF)
