# Supplementary material for: Microalgae as a New Source of Oxylipins: A Comprehensive LC-MS-Based Analysis Using Conventional and Green Extraction Methods
Source: J Agric Food Chem. 2024 Jul 17;72(30):16749–60. doi: 10.1021/acs.jafc.4c03264 (PMC11299188; doi:10.1021/acs.jafc.4c03264)
Supplement: Supplementary file 1 — jf4c03264_si_001.pdf [file jf4c03264_si_001.pdf]

SUPPORTING INFORMATION

Microalgae as a new source of oxylipins: a comprehensive LC-MS based analysis using conventional and green extraction methods

Arturo Auñon-Lopez[1,2], Jon Alberdi-Cedeño[1,3], Marc Pignitter[1,*], Natalia Castejón[4,*]

[1]Institute of Physiological Chemistry, Faculty of Chemistry, University of Vienna, 1090 Vienna, Austria

[2]Vienna Doctoral School in Chemistry (DoSChem), University of Vienna, Währinger Str. 42, 1090 Vienna, Austria

[3]Food Technology, Faculty of Pharmacy, Lascaray Research Center, University of the Basque Country (UPV-EHU), Paseo de la Universidad nº 7, 01006 Vitoria-Gasteiz, Spain

[4]Institute of Food Chemistry and Toxicology, Faculty of Chemistry, University of Vienna, Waehringer Str. 38, 1090 Vienna, Austria

* Equally corresponding authors. E-mail address: marc.pignitter@univie.ac.at (M. Pignitter); natalia.castejon@univie.ac.at (N. Castejón).

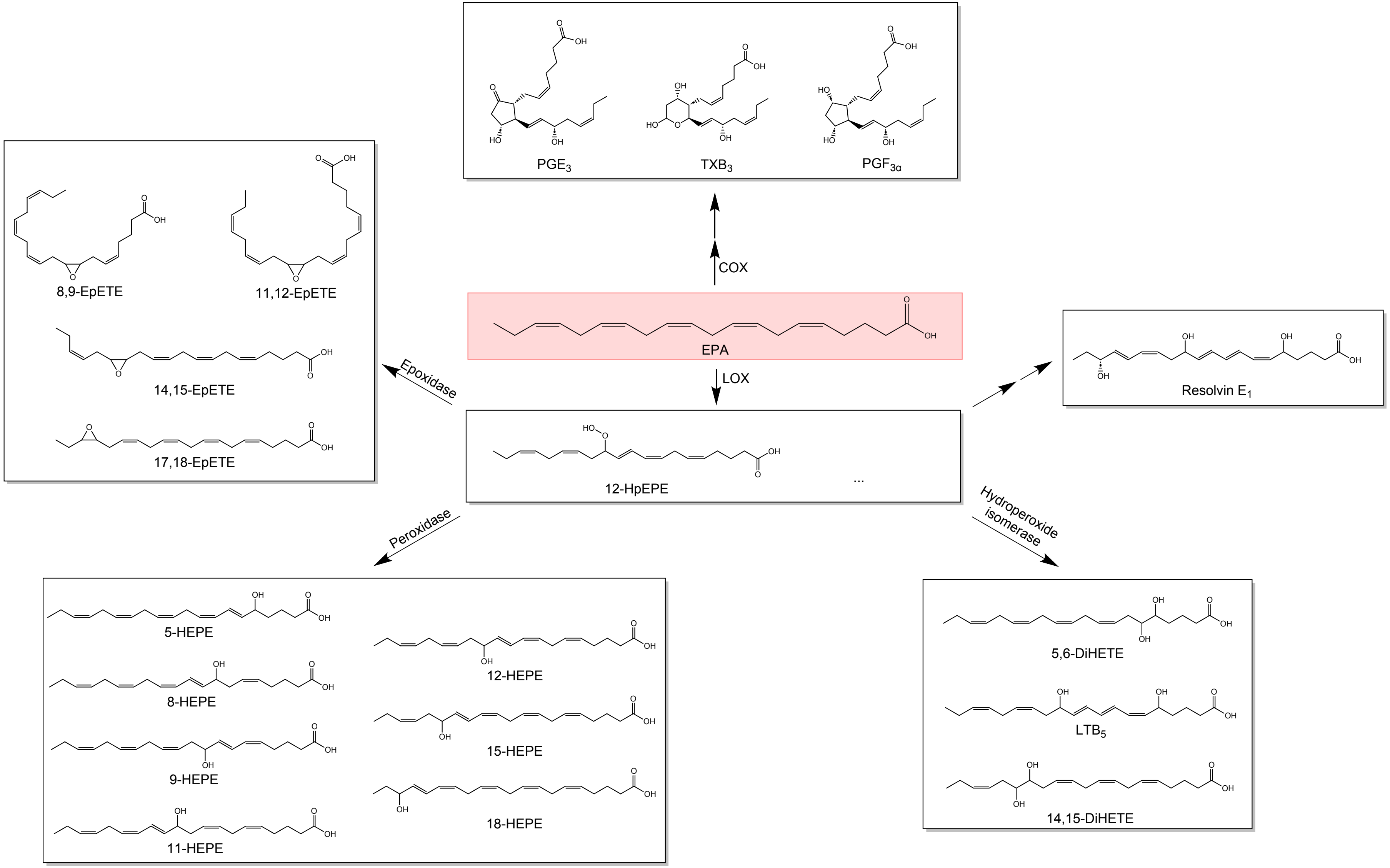

Figure S1. Postulated biosynthetic pathway in microalgae for the EPA-derived oxylipins determined in the present study, Andreou et al., 2009; Gabbs et al.,2015; and Jagusch et al.,2020.[1-3] A "…" symbol represents that the existence of other intermediates of the same class is assumed, but not detected. EPA: Eicosapentaenoic acid; HEPE: Hydroxyeicosapentaenoic acid; EpETE: Epoxyeicosatetraenoic acid; DiHETE: Dihydroxyeicosatetraenoic acid; TX: Thromboxane; PG: Prostaglandin; LT: Leukotriene; HpEPE: Hydroperoxyeicosapentaenoic acid; LOX: Lipoxygenase.

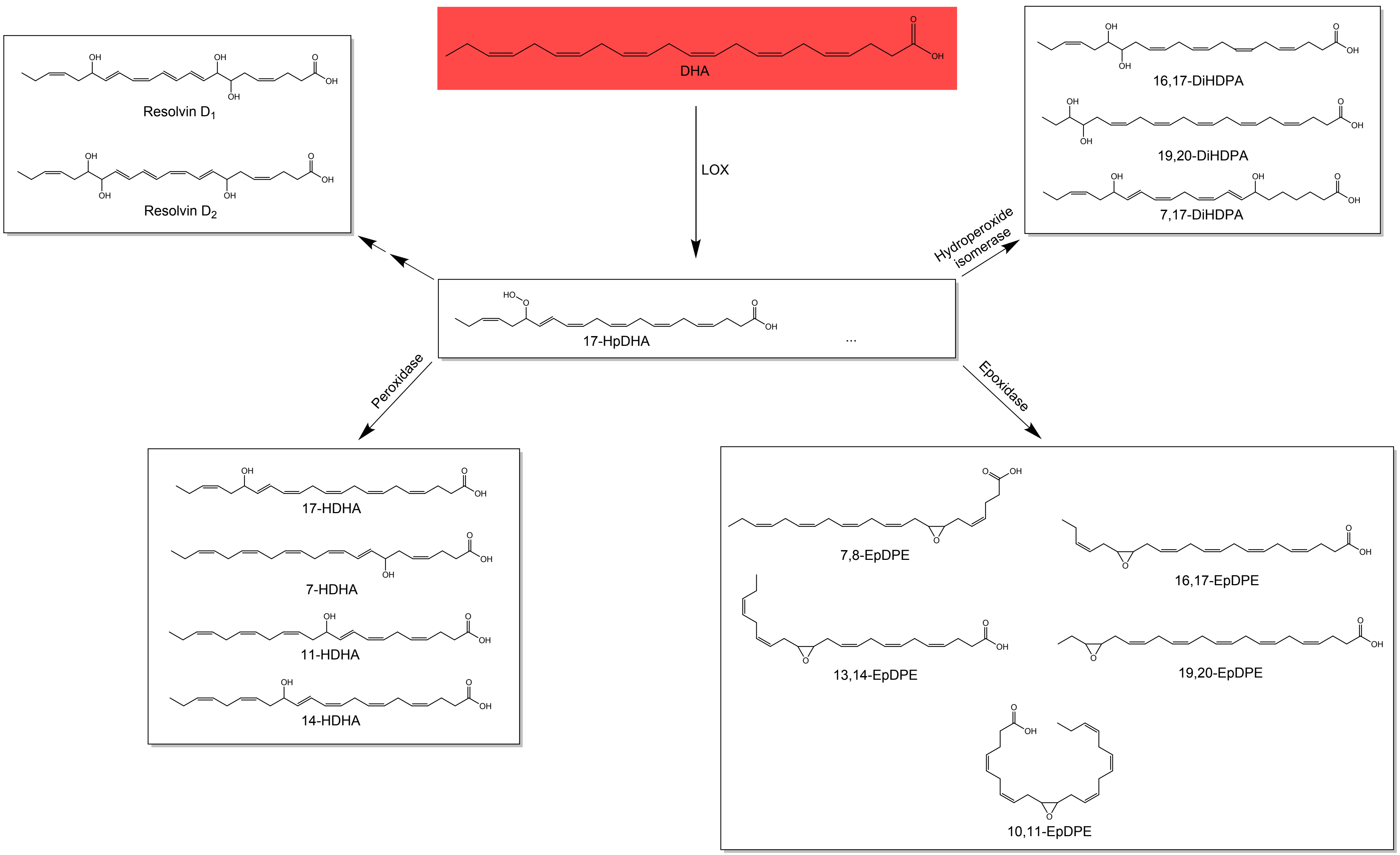

Figure S2. Postulated biosynthetic pathway in microalgae for the DHA-derived oxylipins determined in the present study, based on Andreou et al., 2009; Gabbs et al.,2015; and Jagusch et al.,2020.[1-3] A "…" symbol represents that the existence of other intermediates of the same class is assumed, but not detected. DHA: Docosahexaenoic acid; HDHA: Hydroxydocosahexaenoic acid; EpDPE: Epoxydocosapentanoic acid; DiHDPA: Dihydroxydocosapentaenoic acid; HpDHA: Hydroperoxydocosahexaenoic acid; LOX: Lipoxygenase.

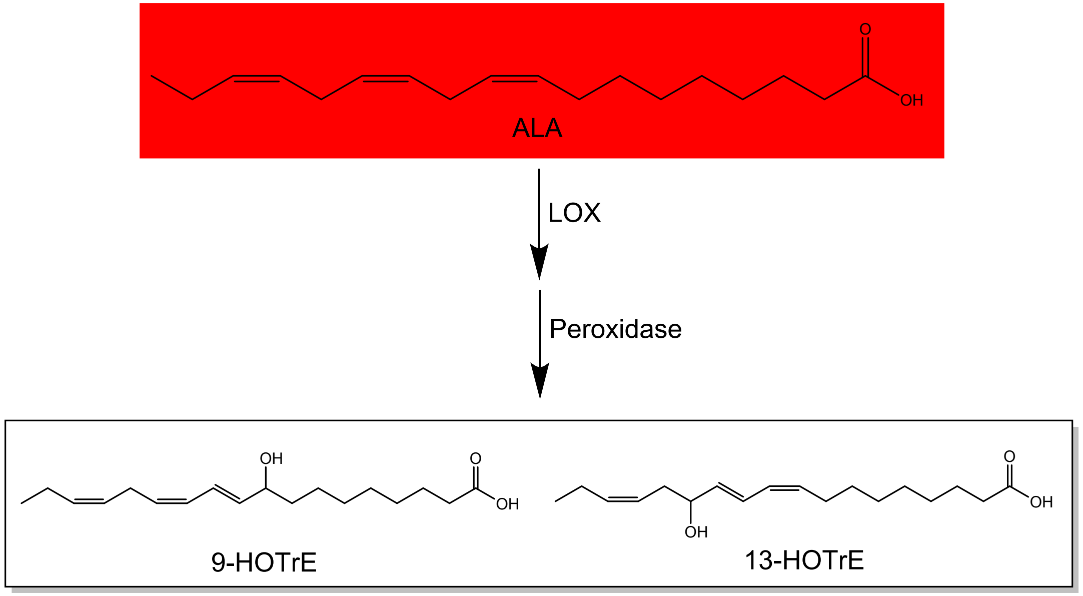

Figure S3. Postulated biosynthetic pathway in microalgae for the ALA-derived oxylipins determined in the present study, based on Andreou et al., 2009; Gabbs et al.,2015; and Jagusch et al.,2020.[1-3] ALA: Linolenic acid; HOTrE: Hydroxyoctadecatrienoic acid; LOX: Lipoxygenase.

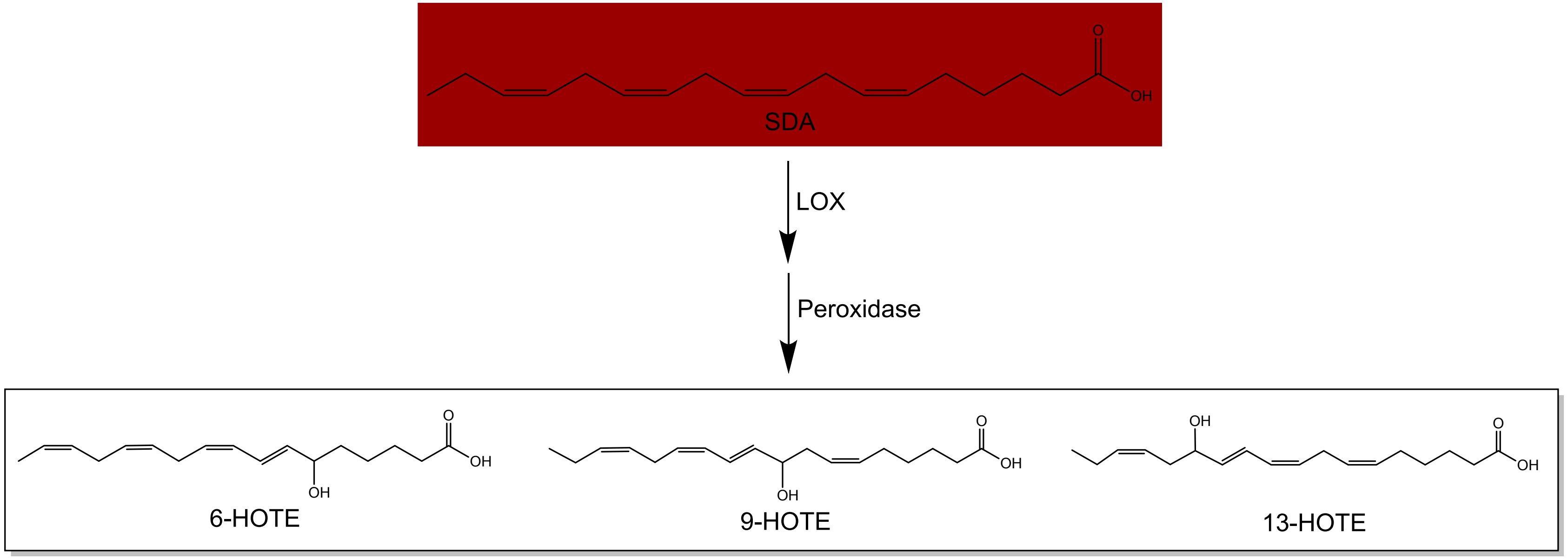

Figure S4. Postulated biosynthetic pathway in microalgae for the SDA-derived oxylipins determined in the present study, Andreou et al., 2009; Gabbs et al.,2015; and Jagusch et al.,2020.[1-3] SDA: Stearidonic acid; HOTE: hydroxyoctadecatetraenoic acid; LOX: Lipoxygenase.

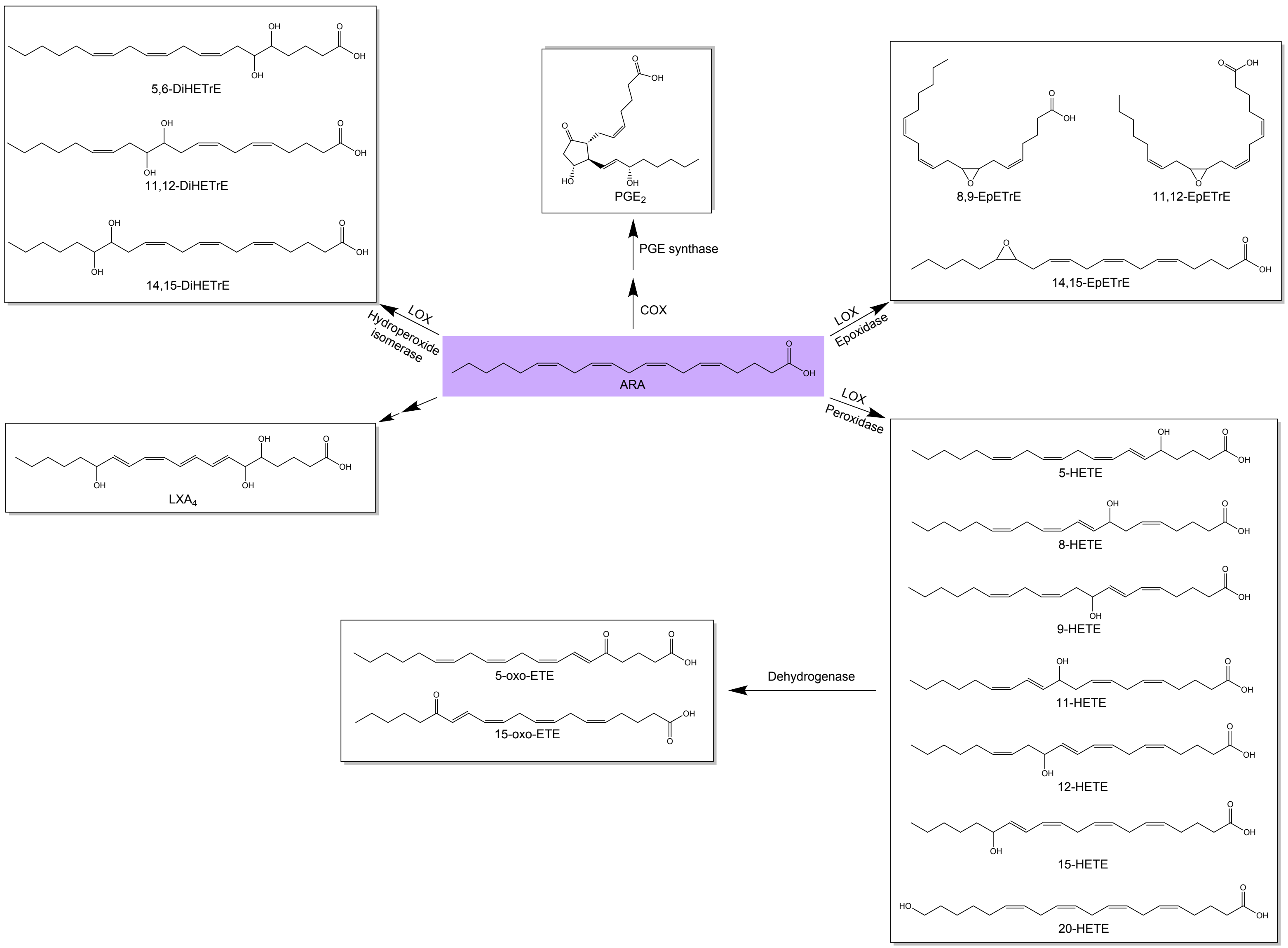

Figure S5. Postulated biosynthetic pathway in microalgae for the ARA-derived oxylipins determined in the present study, Andreou et al., 2009; Gabbs et al.,2015; and Jagusch et al.,2020.[1-3] ARA: Arachidonic acid; HETE: Hydroxyeicosatetraenoic acid; Oxo-ETE: oxoeicosatetraenoic acid; EpETrE: Epoxyeicosatrienoic acid; DiHETrE: Dihydroxyeicosatrienoic acid; LX: Lipoxin; PG: Prostaglandin; LOX: Lipoxygenase; COX: Ciclooxigenase.

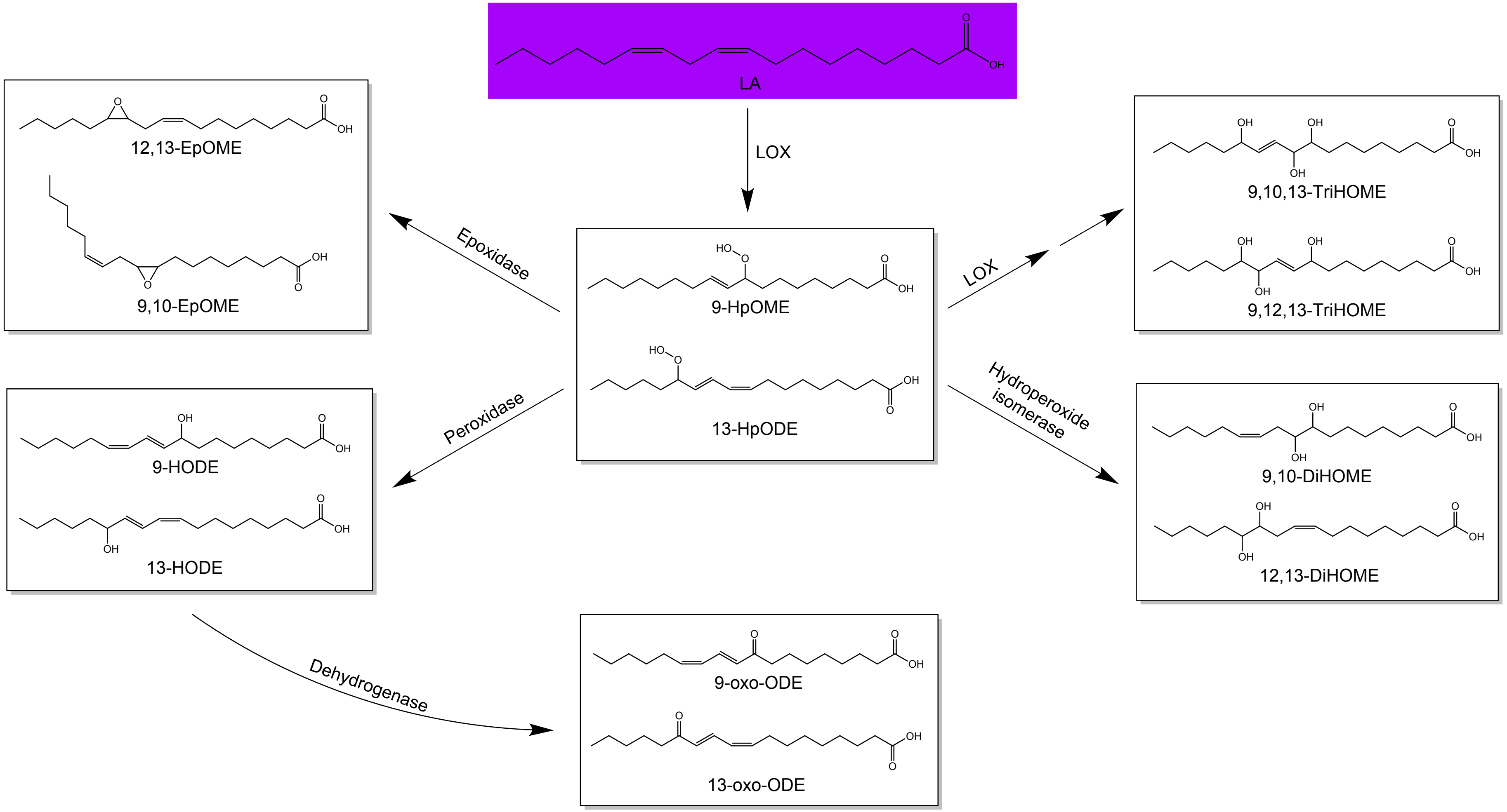

Figure S6. Postulated biosynthetic pathway in microalgae for the LA-derived oxylipins determined in the present study, Andreou et al., 2009; Gabbs et al.,2015; and Jagusch et al.,2020.[1-3] LA: Linoleic acid; EpOME: Epoxyoctadecenoic acid; HOME: Hydroxyoctadecenoic acid; HODE: Hydroxyoctadecadienoic acid; Oxo-ODE: Oxooctadecadienoic acid; HpOME: Hydroperoxyoctadecenoic acid¸ HpODE: Hydroperoxyoctadecadienoic acid; LOX: Lipoxygenase.

Table S1. Free oxylipins analyzed by LC-MS/MS based on previous studies.[4, 5]

precursor fatty acid	free oxylipin	retention time (min)	precursor ion (m/z)	product ion (m/z)*	collision energy (V)
EPA	5-HEPE	23.5	317.2	115.1	4
	8-HEPE	22.5	317.2	155.2	7
	12-HEPE	22.9	317.2	208.0/179.2	4
	15-HEPE	22.0	317.2	247.0/219.2	4
	8;9-EpETE	22.1	317.2	127.2	4
	11;12-EpETE	21.9	317.2	167.2	4
	14;15-EpETE	21.8	317.2	207.2	4
	17;18-EpETE	20.7	317.2	259.2/215.2	10/4
	11-HEPE	22.3	317.2	195.0	10
	9-HEPE	23.1	317.2	149.1/123.1	10
	5,6-DiHETE	18.4	335.2	145.0/115.2	4
	14,15-DiHETE	20.0	335.2	207.2	7
	TXB_3	2.0	367.2	169.1	4
	$PGF_{3\alpha}$	2.0	351.2	193.2	4
	PGE_3	2.0	349.2	269.2	10
	Resolvin E_1	2.1	349.2	195	10
	LTB_5	12.7	333.2	195.1	4
	18-HEPE	21.0	317.2	259.2	10
	12/15-HpEPE	29.4	333.0	271.0	4
DHA	17-HDHA	25.1	343.2	281.2/245.0	4
	7;8-EpDPE	26.1	343.2	141.0/113.1	4
	10;11-EpDPE	25.5	343.2	153.2	4
	13;14-EpDPE	25.3	343.2	193.2	4
	16;17-EpDPE	24.9	343.2	233.2	4
	19;20-EpDPE	24.0	343.2	241.2	7
	16,17-DiHDPA	18.1	343.3	233.1	8
	19,20-DiHDPA	27.8	361.5	273.1	4
	7-HDHA	25.0	361.5	201.2	4

DHA	Resolvin D_1	2.2	343.3	141.0/121.0	10
	Resolvin D_2	2.0	375.2	233.0/175.0	20
	11-HDHA	25.8	359.2	149.0	4
	14-HDHA	25.6	343.3	205.0	4
	17-HpDHA	26.6	359.0	297.0	4
	7,17-DiHDPA	11.2	361.2	263.0	4
ALA	9-HOTrE	20.1	293.2	171.2	4
	13-HOTrE	20.9	293.2	195.1	10
SDA	6-HOTE	18.9	291.2	129.1	4
	9-HOTE	16.7	291.2	171.2	4
	13-HOTE	16.6	291.2	195.2	10
ARA	5-HETE	26.5	319.2	115.1	10
	8-HETE	25.9	319.2	155.2	7
	9-HETE/11-HETE	25.4	319.2	167.2	7
	12-HETE	26.0	319.2	179.2	7
	15-HETE	24.6	319.2	219.2	4
	20-HETE	25.0	319.2	275.1	10
	5-oxo-ETE	28.1	317.2	273.2/203.0	7/17
	15-oxo-ETE	25.7	317.2	113.1	10
	5;6-EpETrE	26.3	319.2	191.1	4
	8;9-EpETrE/11;12-EpETrE	25.1	319.2	167.2	4
	14;15-EpETrE	24.3	319.2	219.3	4
	5,6-DiHETrE	21.7	337.2	145.1	7
	11,12-DiHETrE	18.6	337.2	167.1	13
	14,15-DiHETrE	18.6	337.2	207.1	10
	LXA_4	11	351.2	115.2	10
	PGE_2	2.2	351.2	271.3	16
LA	12;13-EpOME	23.9	295.3	195.2	7
	9;10-EpOME	24	295.3	171.1	7
	9,10-DiHOME	17.5	313.2	201.2	16

LA	12,13-DiHOME	16.6	313.2	183.2	16
	9-HODE	24	295.2	171.1	10
	13-HODE	25.2	295.2	195.2	13
	9-oxo-ODE	25.8	293.2	185.1	13
	13-oxo-ODE	24.5	293.2	195.1	13
	9,10,13-TriHOME	20.4	329.2	171.1	16
	9,12,13-TriHOME	20.8	329.2	211.1	16
	Total HpOME	25	311.1	293.2	10
	13-HpODE	25	311.1	113.1	20
	9-HpOME	25	311.1	123.0	20

EPA: Eicosapentaenoic acid; DHA: Docosahexaenoic acid; ALA: Linolenic acid; SDA: Stearidonic acid; ARA: Arachidonic acid; LA: Linoleic acid; HEPE: Hydroxyeicosapentaenoic acid; EpETE: Epoxyeicosatetraenoic acid; DiHETE: Dihydroxyeicosatetraenoic acid; TX: Thromboxane; PG: Prostaglandin; LT: Leukotriene; HpEPE: Hydroperoxyeicosapentaenoic acid; HDHA: Hydroxydocosahexaenoic acid; EpDPE: Epoxydocosapentanoic acid; DiHDPA: Dihydroxydocosapentaenoic acid; HpDHA: Hydroperoxydocosahexaenoic acid; HOTrE: Hydroxy octadecatrienoic acid; HOTE: hydroxyoctadecatetraenoic acid; HETE: Hydroxyeicosatetraenoic acid; Oxo-ETE: oxoeicosatetraenoic acid; EpETrE: Epoxyeicosatrienoic acid; DiHETrE: Dihydroxyeicosatrienoic acid; LX: Lipoxin; PG: Prostaglandin; EpOME: Epoxyoctadecenoic acid; HOME: Hydroxyoctadecenoic acid; HODE: Hydroxyoctadecadienoic acid; Oxo-ODE: Oxooctadecadienoic acid; HpOME: Hydroperoxyoctadecenoic acid; HpODE: Hydroperoxyoctadecadienoic acid.

Table S2. Omega-3 derived oxylipins from *Microchloropsis gaditana*, *Tisochrysis lutea*, *Phaeodactylum tricornutum* and *Porphyridium cruentum* after Folch extraction.

precursor fatty acid	free oxylipin	concentration (µg/g)			
		M. gaditana	*T. lutea*	*P. tricornutum*	*P. cruentum*
EPA	5-HEPE	156.7 ± 91.8[a]	6.5 ± 5.7[b]	81.0 ± 24.6[ab]	34.0 ± 6.1[b]
	8-HEPE	18.1 ± 10.5[c]	27.8 ± 21.5[c]	304.2 ± 85.6[a]	158.8 ± 16.0[b]
	12-HEPE	5.7 ± 5.1[c]	13.5 ± 9.8[c]	136.8 ± 38.4[a]	72.1 ± 6.1[b]
	15-HEPE	14.0 ± 7.7[c]	21.9 ± 18.3[c]	216.3 ± 58.0[a]	128.9 ± 12.4[b]
	8;9-EpETE	< LOD	0.4 ± 0.3[c]	3.2 ± 0.8[a]	1.6 ± 0.0[b]
	11;12-EpETE	5.7 ± 3.8[c]	9.5 ± 7.3[c]	93.0 ± 24.2[a]	52.1 ± 5.8[b]
	14;15-EpETE	16.2 ± 8.1[c]	28.5 ± 25.2[c]	313.5 ± 91.2[a]	182.3 ± 22.0[b]
	17;18-EpETE	42.1 ± 21.6[c]	65.6 ± 53.0[c]	871.8 ± 257.5[a]	404.0 ± 77.3[b]
	11-HEPE	23.5 ± 13.8[c]	35.7 ± 28.6[c]	375.4 ± 117.1[a]	206.6 ± 23.1[b]
	9-HEPE	48.9 ± 30.0[bc]	30.5 ± 23.9[c]	273.0 ± 84.1[a]	145.2 ± 30.5[b]
	5,6-DiHETE	< LOQ	< LOD	7.8 ± 2.2[a]	2.1 ± 0.1[b]
	14,15-DiHETE	< LOD	< LOD	1.3 ± 0.4[b]	18.2 ± 8.2[a]
	TXB_3	0.7 ± 0.3[b]	1.5 ± 0.7[ab]	0.7 ± 0.5[b]	2.1 ± 0.2[a]
	$PGF_{3\alpha}$	0.1 ± 0.1[b]	0.3 ± 0.2[b]	0.4 ± 0.2[b]	1.7 ± 0.7[a]
	PGE_3	1.9 ± 1.0[b]	3.8 ± 4.4[b]	15.1 ± 7.8[b]	43.0 ± 27.6[a]
	Resolvin E_1	1.3 ± 0.7[c]	1.8 ± 1.0[bc]	4.7 ± 2.1[a]	4.1 ± 1.3[ab]
	LTB_5	14.0 ± 8.5[b]	4.8 ± 3.6[b]	44.7 ± 13.5[a]	41.2 ± 4.5[a]
	18-HEPE	66.0 ± 33.9[c]	102.8 ± 82.9[c]	1367.2 ± 403.9[a]	633.5 ± 121.3[b]
	12/15-HpEPE	6.8 ± 4.8[a]	0.3 ± 0.3[b]	0.1 ± 0.1[b]	0.9 ± 1.1[ab]
DHA	17-HDHA	< LOD	90.0 ± 78.5[a]	10.6 ± 4.2[a]	< LOD
	7;8-EpDPE	< LOD	20.1 ± 18.2[a]	1.7 ± 0.7[a]	< LOD
	10;11-EpDPE	< LOD	30.5 ± 27.6[a]	2.9 ± 1.6[a]	< LOD
	13;14-EpDPE	< LOD	73.5 ± 65.1[a]	9.1 ± 4.2[a]	< LOD
	16;17-EpDPE	< LOD	143.5 ± 125.5[a]	13.6 ± 5.1[a]	< LOD
	19;20-EpDPE	< LOD	204.4 ± 175.5[a]	16.7 ± 6.2[a]	< LOD
	16,17-DiHDPA	< LOD	< LOD	< LOD	< LOD
	19,20-DiHDPA	< LOD	0.5 ± 0.6[a]	< LOD	< LOD

	7-HDHA	< LOD	41.6 ± 37.6^{a}	4.7 ± 1.8^{a}	< LOD
DHA	Resolvin D_1	< LOD	7.7 ± 4.8^{a}	0.8 ± 0.5^{b}	< LOD
	Resolvin D_2	< LOD	25.5 ± 18.7^{a}	2.2 ± 1.1^{b}	< LOD
	11-HDHA	< LOD	31.0 ± 28.4^{a}	3.3 ± 1.5^{a}	< LOD
	14-HDHA	< LOD	37.4 ± 34.2^{a}	4.3 ± 2.6^{a}	< LOD
	17-HpDHA	< LOD	6.2 ± 5.8^{a}	2.0 ± 0.8^{a}	< LOD
	7,17-DiHDPA	< LOD	< LOD	6.3 ± 2.6^{a}	< LOD
ALA	9-HOTrE	2.4 ± 3.4^{a}	52.8 ± 44.4^{a}	4.1 ± 1.0^{a}	< LOD
	13-HOTrE	4.7 ± 5.9^{a}	148.1 ± 122.0^{a}	8.0 ± 1.7^{a}	< LOD
SDA	6-HOTE	< LOD	131.3 ± 109.7^{a}	6.5 ± 1.5^{a}	< LOD
	9-HOTE	< LOD	4.9 ± 2.6^{a}	< LOD	< LOD
	13-HOTE	< LOD	102.5 ± 84.7^{a}	3.2 ± 1.0^{a}	< LOD

Data is shown as mean ± SD ($n \geq 3$). Different lower-case letters (a, b, c) show statistically significant differences ($p < 0.05$). EPA: Eicosapentaenoic acid; DHA: Docosahexaenoic acid; ALA: Linolenic acid; SDA: Stearidonic acid; HEPE: Hydroxyeicosapentaenoic acid; EpETE: Epoxyeicosatetraenoic acid; DiHETE: Dihydroxyeicosatetraenoic acid; TX: Thromboxane; PG: Prostaglandin; LT: Leukotriene; HpEPE: Hydroperoxyeicosapentaenoic acid; HDHA: Hydroxydocosahexaenoic acid; EpDPE: Epoxydocosapentanoic acid; DiHDPA: Dihydroxydocosapentaenoic acid; HpDHA: Hydroperoxydocosahexaenoic acid; HOTrE: Hydroxy octadecatrienoic acid; HOTE: hydroxyoctadecatetraenoic acid; LOD: Limit of detection; LOQ: Limit of quantification.

Table S3. Omega-6 derived oxylipins from *Microchloropsis gaditana*, *Tisochrysis lutea*, *Phaeodactylum tricornutum* and *Porphyridium cruentum* after Folch extraction.

precursor fatty acid	free oxylipin	concentration (µg/g)			
		M. gaditana	*T. lutea*	*P. tricornutum*	*P. cruentum*
ARA	5-HETE	105.8 ± 60.3^{b}	15.9 ± 28.8^{c}	24.9 ± 10.2^{c}	217.6 ± 35.3^{a}
	8-HETE	2.9 ± 1.6^{b}	11.8 ± 22.2^{b}	20.8 ± 9.5^{b}	133.0 ± 28.9^{a}
	9-HETE/11-HETE	3.2 ± 1.2^{b}	15.8 ± 29.9^{b}	22.4 ± 9.9^{b}	199.6 ± 37.8^{a}
	12-HETE	10.0 ± 5.6^{b}	44.8 ± 83.9^{b}	82.1 ± 34.7^{b}	533.0 ± 79.7^{a}
	15-HETE	3.2 ± 1.5^{b}	17.3 ± 32.2^{b}	34.9 ± 12.5^{b}	209.1 ± 16.2^{a}
	20-HETE	< LOD	< LOD	31.8 ± 14.9^{b}	254.2 ± 32.8^{a}
	5-oxo-ETE	13.8 ± 10.1^{b}	< LOD	32.8 ± 26.9^{b}	836.2 ± 118.7^{a}
	15-oxo-ETE	5.8 ± 4.4^{b}	< LOD	38.2 ± 21.0^{b}	386.0 ± 95.0^{a}
	5;6-EpETrE	0.1 ± 0.1^{b}	< LOD	0.4 ± 0.1^{b}	1.9 ± 0.5^{a}
	8;9-EpETrE/11;12-EpETrE	2.0 ± 0.9^{b}	< LOD	14.3 ± 6.3^{b}	127.3 ± 24.1^{a}
	14;15-EpETrE	1.9 ± 0.9^{c}	< LOD	21.5 ± 7.6^{b}	133.1 ± 11.5^{a}
	5,6-DiHETrE	< LOD	< LOD	0.5 ± 0.3^{b}	4.0 ± 1.4^{a}
	11,12-DiHETrE	< LOD	< LOD	< LOD	1.8 ± 0.2^{a}
	14,15-DiHETrE	< LOD	2.9 ± 1.4^{b}	1.5 ± 0.6^{b}	12.2 ± 0.4^{a}
	LXA_4	< LOD	4.0 ± 1.4^{b}	89.1 ± 42.9^{a}	32.2 ± 0.3^{ab}
	PGE_2	1.2 ± 0.5^{b}	5.4 ± 8.0^{b}	4.4 ± 2.5^{b}	38.3 ± 24.8^{a}
LA	12;13-EpOME	1.4 ± 1.1^{c}	19.3 ± 11.0^{ab}	10.5 ± 2.9^{bc}	29.5 ± 3.7^{a}
	9;10-EpOME	0.6 ± 0.2^{c}	13.1 ± 7.5^{ab}	7.0 ± 2.0^{bc}	19.9 ± 2.1^{a}
	9,10-DiHOME	< LOQ	1.5 ± 0.7^{b}	< LOD	7.5 ± 0.3^{a}
	12,13-DiHOME	< LOD	1.8 ± 0.6^{b}	1.9 ± 0.8^{b}	7.0 ± 1.4^{a}
	9-HODE	7.1 ± 6.1^{c}	102.1 ± 58.9^{ab}	53.3 ± 13.9^{bc}	147.4 ± 12.5^{a}
	13-HODE	22.8 ± 18.2^{c}	313.1 ± 176.9^{ab}	176.6 ± 50.5^{bc}	477.9 ± 120.5^{a}
	9-oxo-ODE	9.0 ± 4.6^{c}	68.8 ± 35.4^{b}	56.6 ± 23.2^{b}	127.5 ± 22.8^{a}
	13-oxo-ODE	2.3 ± 1.7^{b}	18.3 ± 10.6^{a}	7.7 ± 3.9^{ab}	17.7 ± 3.1^{ab}
	9,10,13-TriHOME	2.6 ± 1.6^{b}	3.6 ± 2.4^{b}	15.0 ± 4.4^{a}	1.8 ± 0.2^{b}
	9,12,13-TriHOME	< LOD	< LOQ	< LOD	< LOD

	Total HpOME	4.8 ± 4.1[b]	< LOD	< LOD	578.5 ± 231.9[a]
LA	13-HpODE	0.4 ± 0.5[b]	< LOD	< LOD	16.8 ± 6.9[a]
	9-HpOME	< LOD	< LOD	< LOD	1.1 ± 0.5[a]

Data is shown as mean ± SD (n ≥ 3). Different lower-case letters (a, b, c) show statistically significant differences ($p < 0.05$). ARA: Arachidonic acid; LA: Linoleic acid; HETE: Hydroxyeicosatetraenoic acid; Oxo-ETE: oxoeicosatetraenoic acid; EpETrE: Epoxyeicosatrienoic acid; DiHETrE: Dihydroxyeicosatrienoic acid; LX: Lipoxin; PG: Prostaglandin; EpOME: Epoxyoctadecenoic acid; HOME: Hydroxyoctadecenoic acid; HODE: Hydroxyoctadecadienoic acid; Oxo-ODE: Oxooctadecadienoic acid; HpOME: Hydroperoxyoctadecenoic acid; HpODE: Hydroperoxyoctadecadienoic acid; LOD: Limit of detection; LOQ: Limit of quantification.

References

(1) Andreou, A.; Brodhun, F.; Feussner, I. Biosynthesis of oxylipins in non-mammals. *Prog Lipid Res* **2009**, *48* (3-4), 148-170. DOI: 10.1016/j.plipres.2009.02.002.

(2) Gabbs, M.; Leng, S.; Devassy, J. G.; Monirujjaman, M.; Aukema, H. M. Advances in our understanding of oxylipins derived from dietary PUFAs. *Advances in Nutrition* **2015**, *6* (5), 513-540. DOI: 10.3945/an.114.007732.

(3) Jagusch, H.; Baumeister, T. U. H.; Pohnert, G. Mammalian-like inflammatory and pro-resolving oxylipins in marine algae. *ChemBioChem* **2020**, *21* (17), 2419-2424. DOI: 10.1002/cbic.202000178.

(4) Emami, S.; Zhang, Z.; Taha, A. Y. Quantitation of oxylipins in fish and algae oil supplements using optimized hydrolysis procedures and ultra-high performance liquid chromatography coupled to tandem mass-spectrometry. *Journal of Agricultural and Food Chemistry* **2020**, *68* (35), 9329-9344. DOI: 10.1021/acs.jafc.0c02461.

(5) Ostermann, A. I.; Willenberg, I.; Schebb, N. H. Comparison of sample preparation methods for the quantitative analysis of eicosanoids and other oxylipins in plasma by means of LC-MS/MS. *Anal Bioanal Chem* **2015**, *407* (5), 1403-1414. DOI: 10.1007/s00216-014-8377-4.
